# Barriers to Biculturalism: Historical Negation and Symbolic Exclusion Predict Longitudinal Increases in Bicultural Policy Opposition

**DOI:** 10.1177/01461672231209657

**Published:** 2023-11-09

**Authors:** Zoe Bertenshaw, Chris G. Sibley, Danny Osborne

**Affiliations:** 1 University of Auckland, New Zealand

**Keywords:** biculturalism, colonization, ideology, inequality, policy

## Abstract

The colonial ideologies of historical negation and symbolic exclusion (i.e., the “Dark Duo”) promote inequality between settler colonizers and Indigenous peoples by denying the contemporary relevance of past injustices and excluding Indigenous culture from the nation’s identity, respectively. Although their correlates are established, the temporal ordering of the relationship between the Dark Duo and bicultural policy opposition is unclear. We address this oversight by utilizing nine annual waves of panel data from a nationwide random sample of New Zealand adults (*N* = 31,104) to estimate two multigroup RI-CLPMs using the Dark Duo to predict symbolic and resource-based policy opposition (and vice versa). Results revealed that within-person increases in historical negation and symbolic exclusion predicted subsequent increases in symbolic and resource-based bicultural policy opposition for both majority and minority ethnic groups. These relationships were, however, bidirectional, demonstrating a self-perpetuating cycle, whereby the Dark Duo undermines biculturalism and antibiculturalism strengthens the Dark Duo.

With conversations on decolonization gaining traction in recent years, settler colonial nations such as Aotearoa (New Zealand) are in the process of navigating settler colonizer versus Indigenous claims to material and symbolic national resources. Indeed, the persistence of colonial inequities and the continued precarity of Indigenous rights (Human Rights Commission, 2022; United Nations Department of Economic and Social Affairs, 2009) illustrates the dire need for political action that challenges the colonial project. Globally, several steps have been taken to promote Indigenous rights (e.g., the United Nations Declaration on the Rights of Indigenous Peoples; UN General Assembly, 2007). Contemporary Aotearoa (i.e., the location of this study) has similarly seen systematic reforms that better attend to Indigenous needs (e.g., structural changes to advance equity and representation in the political and healthcare systems; Department of Internal Affairs, 2021; Future of Health, 2022). Despite making some headway, Indigenous progress continues to face enduring political obstacles (Mutu, 2018) and active opposition (e.g., see Venutu, 2023; also see Indigenous Missionary Council, 2022). Within this context, it is crucial to understand the factors that shape attitudes toward policies that redress colonial inequities and advance Indigenous rights.

Numerous literatures have explicated the general ideologies that foster support or opposition to racial inequality (e.g., Jost, 2006). For example, system justification theory asserts that system-justifying ideologies maintain unequal systems by fostering perceptions of fairness (Jost & Hunyady, 2005). These ideologies promote political attitudes that reinforce the current system and undermine support for collective action against inequality (Jost et al., 2017). Conservatism embodies a quintessential politically charged, albeit general, ideology with direct links to socio-political outcomes, including support for political parties and policies that reinforce the status quo of intergroup hierarchy (see Jost et al., 2003).

Although general ideologies like conservatism reinforce inequality cross-culturally, a complete understanding of the political power of ideology must also examine belief systems uniquely tailored to specific socio-historical contexts. In settler colonial contexts, culture-specific ideologies maintain racial inequality between the Indigenous community and the settler colonizer group. Sibley (2010) outlines two such ideologies in the Dark Duo Model of colonial^1^ ideology: historical negation and symbolic exclusion. While historical negation asserts that past injustices toward the Indigenous peoples are irrelevant to contemporary inequities, symbolic exclusion contends that the Indigenous cultural identity no longer reflects the nation’s identity.

This study extends the literature on the ideological drivers of inequality by investigating the longitudinal impact of the Dark Duo on opposition to two forms of policies that promote Indigenous-focused biculturalism: resource-based and symbolic-based policies. While resource-based policies directly redistribute resources to disadvantaged groups through legislation (e.g., affirmative action), symbolic-based policies focus on less tangible (though nonetheless important) cultural transformations including formally recognizing Indigenous languages. Despite distinguishing between material and symbolic resources, both types of policies are needed to redress the wrongs of colonization. We thus begin by discussing the origins of historical negation and symbolic exclusion, as well as the functions of these ideologies. We then examine the political implications of the Dark Duo to introduce our hypotheses.

## Settler Colonial Context

Globally, Indigenous groups in colonized nations face disproportionately adverse economic, social, and personal outcomes (see González et al., 2022). These inequities occur within settler colonial societies because the structural systems introduced by the colonizers represent their values and inherently clash with the Indigenous communities upon which they are forced (Reid et al., 2017). It is thus unsurprising that these systems favor settler colonizer groups while undermining the material, physical, and mental well-being of Indigenous peoples. For example, global statistics demonstrate lower levels of income, employment, education, mental health, and physical health as well as elevated rates of child mortality, poverty, incarceration, drug and alcohol abuse, and suicide for Indigenous groups (for a systematic review, see González et al., 2022). This alarming pattern also emerges in Aotearoa, with Māori incurring various hardships across myriad social well-being indicators (Ministry of Social Development, 2016). These shared adverse outcomes underscore the universal trauma of colonization faced by Indigenous peoples.

Although general ideologies reinforce the status quo in most societies, settler colonial nations have a unique socio-historical context that requires distinct belief systems to justify race-based inequalities. As Sibley (2010) notes, most settler colonial societies have a well-documented history of injustices waged against the Indigenous communities who have an irrefutable belongingness to the nation. Indeed, documentation of colonization renders past injustices faced by Indigenous peoples indisputable. So, too, is the nationality of Indigenous peoples who were “here” precolonization. These incontrovertible facts invalidate other general ideologies used to justify inequities against noncolonized ethnic minorities (e.g., nationalism). For example, in cultures that disadvantage recent immigrant groups, inequality is often justified by ideologies asserting that such groups “do not belong” and, thus, are not entitled to the nation’s resources. Because it is implausible to attribute foreigner status to Indigenous peoples, settler colonial societies develop culture-specific belief systems to maintain and justify inequalities between the settler colonizers and the colonized.

## Dark Duo Functions

When past injustices are undeniable, historical negation justifies the settler colonizer group’s advantage by acknowledging past wrongdoings but asserting that they are irrelevant to contemporary inequalities (see Sibley, 2010). As such, Indigenous claims for reparations are undermined by separating the past and present and by denying the intergenerational impacts of colonization. Similarly, symbolic exclusion sustains the advantage of the majority group by framing Indigenous culture as irrelevant to the national prototype. Specifically, symbolic exclusion centralizes the identity of the nation around characteristics of the settler colonizers and rejects Indigenous culture, such as language and practices, from mainstream society. Because the criterion for judgment in social structures is based on the norms of that society, categorizing a group as nonnormative encourages discrimination toward that group while advantaging those who are seen as normative (Mummendey & Wenzel, 1999). Accordingly, symbolic exclusion is a powerful ideological tool used to subvert Indigenous peoples’ social status and rights within their home country.

Despite being distinct, albeit correlated, belief systems, the Dark Duo work together to legitimize the dominant status of the settler colonizers. Specifically, the Dark Duo Model theorizes that these ideologies minimize settler colonizers’ obligations to equally distribute national resources, thus bolstering their social and material privileges (Sibley, 2010). Given these dangerous possibilities, it is crucial to assess the actual impact of the Dark Duo on myriad political attitudes.

## Political Implications

In democratic contexts, citizens’ political choices bridge the gap between ideologies and social outcomes. Past literature examines the political routes through which the Dark Duo maintains racial inequalities in settler colonial nations, including political party alignment, attitudes toward progressive collective action, and, more directly, bicultural policy support. For example, Sibley (2010) revealed that historical negation and symbolic exclusion both independently correlated positively with support for the conservative political party in Aotearoa, but negatively with support for the liberal party (see also Sibley & Osborne, 2016). Greaves and colleagues (2014) further identified bidirectional cross-lagged effects between the Dark Duo and political party preferences, indicating that historical negation and symbolic exclusion both elevate and are elevated by conservative party preferences.

In addition to weakening support for progressive political parties, the Dark Duo undermines support for social change. Osborne and colleagues (2017) examined the longitudinal relationship between the Dark Duo and collective action support. Their analyses revealed unidirectional, cross-lagged effects whereby both historical negation and symbolic exclusion predicted decreased support for protests on behalf of Māori rights. Together, these results indicate that the Dark Duo fosters anti-egalitarian outcomes in general. However, because party votes and protest do not necessarily translate into policies that advance Indigenous rights, we must examine the relationship between the Dark Duo and bicultural policy support specifically to determine the direct route between colonial ideologies and specific forms of colonial inequality.

Providing some initial insights into this relationship, cross-sectional studies reveal that both historical negation and symbolic exclusion correlate negatively with support for resource-based bicultural policies (Newton et al., 2018; Satherley & Sibley, 2018; Sibley et al., 2008). Although these correlations establish a relationship, they cannot speak to the direction of these associations. Indeed, research has yet to examine the temporal ordering of the relationship between symbolic exclusion and support for resource- and symbolic-based bicultural policies—necessitating a longitudinal investigation of these relationships.

Two papers do, however, provide preliminary insights into the directional association between historical negation and both policies among the settler colonizer group. First, Sibley and colleagues (2008) revealed that students primed with historical negation increased their opposition to resource-based policies (but not symbolic-based policies). Second, Sibley and Liu (2012) ran two separate longitudinal studies with student samples and found that, across both studies, historical negation predicted later increases in opposition to resource-based (but not symbolic-based) policies. Resource-based policy opposition did not, however, predict changes in historical negation. Interestingly, symbolic policy opposition predicted historical negation in one study, but not the other.

Collectively, the literature demonstrates that historical negation predicts opposition to resource-based policies, while the relationship between historical negation and symbolic policy support is unclear. The discrepancies across studies may be due to the relatively small sample sizes used (*N*s = 100–200), which can produce spurious results. In addition, Sibley and Liu (2012) used student samples, which limits the generalizability of their results (see Henry, 2008). Finally, and perhaps most critically, the only longitudinal work to date that examines the temporal ordering of the relationship between historical negation and policy preferences utilized traditional cross-lagged panel models (CLPMs; Sibley and Liu, 2012). Yet, traditional CLPMs have recently been critiqued for confounding between- and within-person processes (see Berry & Willoughby, 2017; Hamaker et al., 2015; Osborne & Little, in press). Thus, traditional CLPMs cannot illustrate the impact changes within a person’s ideological views have on later policy positions.

To gain insight into the temporal ordering of the Dark Duo and policy support, we must separate the between-person effects (i.e., stable trait-like differences between people that persist over time) from the within-person effects (i.e., departures from an individual’s trait level at a specific assessment occasion) that form the focus of psychological theory. Fortunately, the recently developed random intercept CLPM (RI-CLPM) allows researchers to distinguish between these two distinct processes (for a detailed overview of this modeling approach, see Hamaker et al., 2015; Osborne & Little, in press). Thus, a RI-CLPM presents the first opportunity to examine whether a departure from a person’s trait-level endorsement of the Dark Duo at one-time point predicts a departure from their trait-level policy preferences at a subsequent time point. By directly evaluating the relationship between within-person fluctuations in ideology and policy preferences year-to-year, we can provide the strongest test of the temporal ordering of the Dark Duo and bicultural policy support to date.

## The Current Study

This study seizes this unique opportunity and addresses the oversights of past research by investigating the within-person temporal associations historical negation and symbolic exclusion have with resource- and symbolic-based policy opposition. To these ends, we use nine annual waves of longitudinal panel data from a nationwide random sample of adults in Aotearoa to examine the cross-lagged effects of the Dark Duo on policy opposition and vice versa. Given the vast structural and social inequities between Pākehā (New Zealand Europeans; the settler colonizers) and Māori (Ministry of Social Development, 2016; Reid et al., 2017), Aotearoa offers a constructive and critical setting for this research.

Past literature has focused on the political function of the Dark Duo among settler colonizers, given these ideologies reinforce their privilege (e.g., see Sibley & Liu, 2012; Sibley et al., 2008). However, historical negation and symbolic exclusion also operate among ethnic minorities, including Indigenous peoples (Sibley, 2010; Sibley & Osborne, 2016). Although system-justifying ideologies contravene the interests of groups disadvantaged by the system, they can also provide palliative benefits (Bahamondes et al., 2019; Sengupta et al., 2017; Vargas-Salfate, 2019; for a review, see Vargas-Salfate et al., 2018). Specifically, to alleviate the negative emotional consequences of acknowledging the illegitimacy of their disadvantaged status, members of structurally deprived groups may develop a sense of false consciousness around the fairness of the system by endorsing system-justifying ideologies (see Jost & Hunyady, 2003). Thus, although the motivation differs (e.g., see Bahamondes et al., 2022), justification of the system is likely to be (at least somewhat) present among both advantaged and disadvantaged groups. As such, it is important to evaluate the political function of the Dark Duo among both groups.

Given the role system justification plays in system maintenance (Jost et al., 2017; Jost & Hunyady, 2005), system-justifying ideologies like the Dark Duo should undermine support for equality-oriented policies. Indeed, by separating colonial injustices from the present and excluding the Indigenous culture from the nation’s identity, historical negation and symbolic exclusion minimize Indigenous peoples’ rights to reparations and national resources, thereby justifying colonial inequities (Sibley, 2010). Through this ideological legitimation, the Dark Duo should foster opposition to the redistribution of both symbolic and material resources. Given the link between (anti-)bicultural ideology and (anti-)bicultural policy, coupled with research demonstrating system justification processes within disadvantaged groups (e.g., Osborne et al., 2017), we expect the legitimizing process between the Dark Duo and bicultural policy opposition to emerge among both ethnic majority and minority groups.

Although we expect to see similar processes unfold across groups, we must allow space for different associations to present. Indeed, bicultural policies have contrasting implications for the settler colonizers and Indigenous peoples—whereas bicultural policies wrestle away power from settler colonizers, they advance the rights of Indigenous peoples. As system-maintaining policies pose more of a direct realistic threat for Indigenous peoples than do abstract ideologies, endorsement of the Dark Duo may *not* translate to bicultural policy opposition among Māori. Thus, it is necessary to examine these processes separately for those who are supported vs disadvantaged by the status quo of coloniality.

Another reason to examine these processes separately across groups focuses on the onus of responsibility for redressing existing inequities. Specifically, settler colonizers have both the capacity and the moral responsibility to challenge the myriad colonial inequities that maintain their resource and symbolic advantage. Indeed, the majority group has momentous political power given their social status, resources, and population size (estimated at 70.2% of the population as per the 2018 Census; Stats NZ, 2020). Thus, settler colonizers represent both the most accessible aid and obstacle to implementing bicultural policies—necessitating a robust understanding of the factors that guide their support for these policies, specifically. For these reasons, we assess the relationship between ideology and policy preference independently for the ethnic majority group (Pākehā) and ethnic minorities (inclusive of Māori).

In sum, because the Dark Duo legitimizes colonial inequities, we predict the following: Within-person increases in historical negation at an initial assessment should predict within-person increases in resource-based policy opposition at the following assessment for both the ethnic majority group (Hypothesis 1a) and ethnic minorities (Hypothesis 1b). Similarly, within-person increases in symbolic exclusion at an initial assessment should predict within-person increases in opposition to symbolic policies at the following assessment for the ethnic majority group (Hypothesis 2a) and ethnic minorities (Hypothesis 2b). We also examine the possibility that these associations are bidirectional by incorporating reciprocal cross-lagged effects into our modeling framework.

## Method

### Data Access

Our data are part of the New Zealand Attitudes and Values Study (NZAVS). Full copies of the NZAVS data files are held by all members of the NZAVS management team and advisory board. A de-identified data set containing the variables analyzed in this manuscript is available upon request from the corresponding author or any NZAVS advisory board member for the purpose of replicating or checking our results. Surveys for each of the nine annual waves of the study, as well as the *Mplus* syntax and correlation matrices used to run these analyses, are also available on OSF: https://osf.io/x93f8/?view_only=be3128ff766c4d2baf9c21356d06b9ce. Although we did not preregister our hypotheses, our predictions originate from the Dark Duo research program which began in 2010. Indeed, Sibley (2010) initially theorized that the Dark Duo undermined support for biculturalism. We aim to provide the first comprehensive test of this hypothesis here. We report all exclusions in the study. The complete NZAVS survey is available here: https://osf.io/75snb/.

### Sampling Procedure

We analyzed data from Times 1–9 of the NZAVS—a longitudinal national probability study of adults that began in 2009. Sampling for the NZAVS occurred on five occasions. In 2009 (Time 1), a random sample of adults from the electoral roll were invited to participate in a projected 20-year longitudinal panel study. This first sampling occasion yielded 6,518 participants (response rate = 16.6%). By 2011, 3,918 participants remained in the study (60.1% retention from Time 1). To address sample attrition, a nonrandom booster sample was recruited from the website of a nationwide newspaper. This second sampling occasion yielded 2,966 new participants and increased the sample size at Time 3 to 6,884 participants.

To further increase the size and diversity of the sample, we conducted three additional booster samples by randomly sampling (without replacement) the electoral roll, oversampling hard-to-reach populations. The first of these three sampling occasions was in 2012 (Time 4) and used multiple sample frames to recruit 5,107 new participants into the study (response rate = 10.0%), plus an additional 265 unmatched participants or unsolicited opt-ins. The second sampling occasion occurred in 2013 (Time 5) and recruited 7,579 new participants into the study (response rate = 10.6%), whereas the third sampling occasion occurred in 2016 (Time 8) and recruited 7,667 new participants into the study (response rate = 9.6%). Thus, Time 8 included 21,936 participants (13,781 retained from a prior time point, 7,667 additions from booster sampling, and 488 unmatched or unsolicited opt-ins). By 2017 (Time 9), 17,072 participants remained in the study (retention rate from Time 8 = 72.0%). In total, 31,545 participants completed at least one of the nine waves of the study.

### Participants

Of these 31,545 participants, 31,104 (i.e., 98.6% of the full sample) completed our variables of interest and formed the basis of the sample for this study. To examine the replicability of our results across structurally advantaged and disadvantaged groups, analyses were grouped by ethnic majority and minority group status. The ethnic majority group consisted of 23,166 Pākehā/New Zealand Europeans (*M*_age at Time 1_ = 43.34, *SD* = 14.59; 61.3% women, 38.5% men, 0.2% gender diverse). Due to the sample size requirements for our analyses, the ethnic minority group included all ethnic minorities in Aotearoa. Thus, the ethnic minority group consisted of 7,938 individuals (*M*_age at Time 1_ = 39.27, *SD* = 13.79; 64.2% women, 35.5% men, 0.3% gender diverse) including Māori (*N* = 5,127), Pacific Islanders (*N* = 1,133), and Asians (*N* = 1,678).

### Measures

This study measured historical negation, symbolic exclusion, resource policy opposition, and symbolic policy opposition at nine consecutive annual waves. Unless noted, items were rated on a 7-point Likert-type scale from 1 (*strongly disagree*) to 7 (*strongly agree*). Tables S1a (ethnic majority) and S1b (ethnic minority) in the Online Supplement display the descriptive statistics and bivariate correlations for the means of these four variables across all nine time points.

*Historical Negation* was assessed using three items from Sibley et al. (2008): “We should all move on as one nation and forget about past differences and conflicts between ethnic groups,” “We should not have to pay for the mistakes of our ancestors,” and “People who weren’t around in previous centuries should not feel accountable for the actions of their ancestors” (Majority group α = .80 - .85; Minority group α = .76 - .81). Items were averaged together for each group at each assessment occasion so that higher values reflect higher levels of historical negation.

*Symbolic Exclusion* was assessed using three items from Sibley (2010): “I think that Māori culture helps to define New Zealand in positive ways” (reverse-coded), “I reckon Māori culture should stay where it belongs—with Māori. It doesn’t concern other NZers,” and “New Zealand would be a better place to live if we forgot about trying to promote Māori culture to everyone” (Majority group α = .83—.87; Minority group α = .72—.81). After reverse-scoring the first item, items were averaged together for each group at each assessment occasion so that higher values reflect higher levels of symbolic exclusion.

*Resource Policy Opposition* was assessed using four items from Liu and Sibley (2006). Participants rated their support for the following policies: “Māori ownership of the seabed and foreshore,” “Reserving places for Māori students to study medicine,” “Rates exemptions on Māori land,” and “Crown (government) ownership of the seabed and foreshore.” Items were rated on a 7-point Likert-type scale from 1 (Strongly Oppose) to 7 (Strongly Support; Majority group α = .76 - .81; Minority group α = .82 - .84). After reverse-scoring all four items, items were averaged together for each group at each assessment occasion so that higher values reflect greater resource policy opposition.

*Symbolic Policy Opposition* was assessed using four items from Liu and Sibley (2006). Participants rated their support for the following policies: “Performance of the Haka at international sports events,” “Waitangi Day as a national celebration of biculturalism,” “Teaching Māori language in New Zealand primary schools,” and “Singing the national anthem in Māori and English.” Items were rated on a 7-point Likert-type scale from 1 (*strongly oppose*) to 7 (*strongly support*; Majority group α = .77–.80; Minority group α = .73–.76). All four items were reverse-scored and averaged together for each group at each assessment occasion so that higher values reflect greater symbolic policy opposition.

## Results

### Preliminary Analyses

Before testing our hypotheses, we examined the measurement invariance of our four multi-item measures both across all nine assessment occasions and between the majority and minority groups (for our syntax, see OSF). Measurement invariance is a critical first step in longitudinal and/or multigroup analyses (see Milfont & Fischer, 2010), and involves the estimation of three increasingly restrictive measurement models that investigate configural (i.e., identical factor loading patterns), metric (i.e., equally sized congeneric factor loadings), and scalar (i.e., equally sized congeneric item intercepts) invariance. Because noninvariance implies that the meaning of a construct varies across groups and/or time, (at least) metric invariance is needed for meaningful between-group and/or across time comparisons (see Putnick & Bornstein, 2016).

To assess measurement invariance in this study, we estimated 18 confirmatory factor analyses in *Mplus* Version 8.9 (Muthén & Muthén, 1998-2017) using full information maximum likelihood (FIML) estimates to address missing data (see Enders & Bandalos, 2001). We began by examining the longitudinal measurement invariance of the Dark Duo and policy opposition in minority and majority groups separately. Starting with the Dark Duo in the minority group,^2^ we estimated a configural invariant measurement model in which the three items that comprised historical negation at Time 1 were the same three items that comprised historical negation at Times 2 to 9. Similarly, the three items that comprised symbolic exclusion at Time 1 were the same three items that comprised symbolic exclusion at Times 2 to 9.

Table 1 demonstrates that the configural invariant model fit these data well, χ2 _(1009)_ = 2,184.494, comparative fit index (CFI) = .983, root mean square error of approximation (RMSEA) = .012 [.012, .013], standardized root mean square residual (SRMR) = .052. As such, we estimated a more restrictive metric invariant model by constraining the congeneric factor loadings for each latent construct to equality across time (e.g., the first item factor loading for historical negation at Time 1 was constrained to be equal to the first item factor loading for historical negation at Times 2–9). We placed analogous constraints on the factor loadings for symbolic exclusion. Using Cheung and Rensvold’s (2002) criterion to compare the fit between two increasingly restrictive measurement models (i.e., ΔCFI < .010), our model displayed metric invariance. We therefore estimated a scalar invariant model by adding equality constraints to congeneric item intercepts (e.g., the first item intercept for historical negation at Time 1 was constrained to be equal to the first item intercept for historical negation at Times 2–9). Because these additional constraints did not reduce model fit relative to the metric invariant model, the Dark Duo displayed scalar invariance across time for the minority group. Identical steps were taken to examine the longitudinal measurement invariance of the Dark Duo for the majority group, revealing that the Dark Duo was also scalar invariant across time (Table 1). We therefore conducted a multigroup analysis of the longitudinal measurement invariance of the Dark Duo, beginning with a configural invariant model. We then constrained to equality the congeneric factor loadings both across time and between groups to estimate a metric invariant measurement model. After achieving metric invariance, we further constrained to equality the congeneric item intercepts both across time and between groups. Because the ΔCFI from the metric to scalar invariant models was greater than .010, we were unable to accept the model with scalar invariance and cannot interpret mean differences in the Dark Duo over time between the minority and the majority groups. Nevertheless, we can proceed with the focal aim of this study and assess the relationships between variables across both time and groups because our model demonstrated the requisite metric invariance.

**Table 1:** Fit Statistics for the Multigroup Measurement Models Estimated Using Maximum Likelihood With Robust Estimates.

	χ^2^	*df*	RMSEA	RMSEA 90% CI	SRMR	CFI	∆CFI	Pass
Dark Duo								
Minority Group (*N* = 7,669)								
1. Configural invariance	2,184.494***	1,009	.012	[.012, .013]	.052	.983	—	—
2. Metric invariance	2,355.941***	1,041	.013	[.012, .014]	.051	.981	.002	Yes
3. Scalar invariance	2,851.163***	1,073	.015	[.014, .015]	.052	.974	.007	Yes
Majority Group (*N* = 23,063)								
1. Configural invariance	4,240.962***	1,009	.012	[.011, .012]	.032	.990	—	—
2. Metric invariance	5,067.467***	1,041	.013	[.013, .013]	.031	.987	.003	Yes
3. Scalar invariance	7,462.819***	1,073	.016	[.016, .016]	.034	.980	.007	Yes
Multigroup Analyses (30,732)								
1. Configural invariance	6,390.899***	2,018	.012	[.012, .012]	.038	.989	—	—
2. Metric invariance	7,448.266***	2086	.013	[.013, .013]	.038	.986	.003	Yes
3. Scalar invariance	12,617.181***	2,170	.018	[.017, .018]	.045	.973	.013	No
Policy Opposition								
Minority Group (*N* = 7,926)								
1. Configural invariance	4,498.549***	2,043	.012	[.012, .013]	.044	.981	—	—
2. Metric invariance	4,649.838***	2,091	.012	[.012, .012]	.045	.980	.001	Yes
3. Scalar invariance	5,339.445***	2,147	.014	[.013, .014]	.046	.975	.005	Yes
Majority Group (*N* = 23,139)								
1. Configural invariance	12,511.347***	2,043	.015	[.015, .015]	.053	.977	—	—
2. Metric invariance	13,003.366***	2,091	.015	[.015, .015]	.054	.976	.001	Yes
3. Scalar invariance	16,363.874***	2,147	.017	[.017, .017]	.054	.969	.007	Yes
Multigroup Analyses (31,065)								
1. Configural invariance	16,874.917***	4,086	.014	[.014, .014]	.051	.978	—	—
2. Metric invariance	18,158.210***	4,189	.015	[.014, .015]	.059	.976	.002	Yes
3. Scalar invariance	25,408.073***	4,306	.018	[.018, .018]	.061	.964	.012	No

*Note*. Configural (same factor loading patterns), metric (equal congeneric factor loadings), and scalar (equal congeneric intercepts) models were estimated sequentially. RMSEA = root mean square error of approximation; CI = confidence interval; CFI = comparative fit index; SRMR = standardized root mean square residual.

*** *p* ≤ .001.

We followed the same steps to examine the longitudinal measurement invariance for resource and symbolic policy opposition. Table 1 indicates that resource and symbolic policy opposition were scalar invariant across time within both the majority and minority groups. Yet only metric invariance was held for the multigroup longitudinal measurement model for policy opposition. Thus, we are unable to interpret mean-level changes in these constructs across groups but can confidently assess our focal hypotheses regarding the relationships between the Dark Duo and policy opposition across both time and groups.

### Overview of Focal Analyses

Given its attempt to divorce colonial injustices from current inequities, historical negation should predict within-person increases in opposition to resource-based policies over time for both the ethnic majority group (Hypothesis 1a) and ethnic minorities (Hypothesis 1b). Similarly, given its aim to reject the Indigenous culture from the nation’s identity, symbolic exclusion should predict within-person increases in opposition to symbolic policies over time for both the ethnic majority group (Hypothesis 2a) and ethnic minorities (Hypothesis 2b).

To test these hypotheses, we followed Mulder and Hamaker’s (2021) approach and estimated two separate (i.e., one for resource policy opposition and one for symbolic policy opposition) multigroup RI-CLPMs in *Mplus version* 8.9 (Muthén & Muthén, 1998-2017) using minority status as the grouping variable and FIML to handle missing data. Due to complications^3^ from having 54 latent variables in each of these models (i.e., 3 constructs × 9 assessments × 2 groups), we used the mean scores of each variable at each assessment occasion (instead of latent variables) to estimate our RI-CLPMs. Specifically, random intercepts (which reflect stable between-person differences) were estimated by fixing to 1 the factor loadings for each mean-scaled manifest variable at each assessment occasion. To capture the within-person deviations from these cross-time means, we simultaneously estimated a new latent variable for each manifest variable at each assessment occasion by constraining the factor loading and residual variance of each mean-scaled variable at each assessment occasion to 1 and 0, respectively.

After partitioning the variance in participants’ responses into between-person and within-person effects, we estimated the within-person longitudinal associations between the Dark Duo and opposition to the given policy by regressing the within-person latent variables at Time 9 onto the corresponding within-person variables at Time 8 and so on. In doing so, we estimated both models as a nonstationary process by allowing the autoregressive and cross-lagged effects to vary across assessment occasions. Because there was no reason to expect the size of the autoregressive and cross-lagged associations to systematically vary across assessment occasions, we then estimated both models as stationary processes within each group. For example, the autoregressive effect of historical negation at Time 1 on historical negation at Time 2 for ethnic majorities was constrained to equal the autoregressive effect of historical negation at Time 2 on historical negation at Time 3 for ethnic majorities and so on. Because model fit significantly declined for both models (see below), we estimated two final multigroup RI-CLPMs with partial stationarity by constraining to equality all the congeneric autoregressive and cross-lagged effects that were statistically similar in size but freely estimating all paths that reduced model fit when constrained to equality (see Osborne & Little, in press).

### Resource Policy Opposition

Our nonstationary model including the Dark Duo and resource policy opposition provided an excellent fit to these data, χ^2^_(492)_ = 3,222.666, CFI = .992, RMSEA = .019 [.018, .020], SRMR = .049. Although the fully stationary model also fit these data well, χ^2^_(618)_ = 4137.996, CFI = .990, RMSEA = .019 [.019, .020], SRMR = .053, constraining every congeneric path to equality significantly reduced model fit, Δ^2^_(126)_ = 915.330, *p* < .001. As such, we estimated a partially stationary model that only constrained to equality congeneric paths that did not reduce model fit. This sequential process produced a partially stationary model that fit these data well, χ^2^_(574)_ = 3,269.158, CFI = .992, RMSEA = .017 [.017, .018], SRMR = .049, and did not significantly differ from the nonstationary model, Δ^2^_(82)_ = 46.498, *p* = .999. Because this model provides a more parsimonious account of our data than does the nonstationary model without compromising model fit, we report the unstandardized regression coefficients for our model with partial stationarity below.

Results for the ethnic majority group revealed that the three random intercepts correlated positively with each other. Specifically, the random intercept for historical negation correlated positively with the random intercept for symbolic exclusion (*b* = 1.05, 95% confidence interval [CI]: [1.02, 1.08]; *p* < .001), indicating that those who tended to be high on historical negation across all nine assessment occasions also tended to be high on symbolic exclusion. Between-person differences in historical negation (*b* = 1.18, 95% CI [1.15, 1.21]; *p* < .001) and symbolic exclusion (*b* = 1.08, 95% CI [1.05, 1.11]; *p* < .001) also correlated positively with resource policy opposition. Constraining to equality the correlations the Dark Duo had with resource policy opposition significantly reduced model fit, Δ^2^_(1)_ = 80.511, *p* < .001, indicating that historical negation was a stronger correlate than symbolic exclusion of resource policy opposition at the between-person level of analysis.

**Figure 1:**
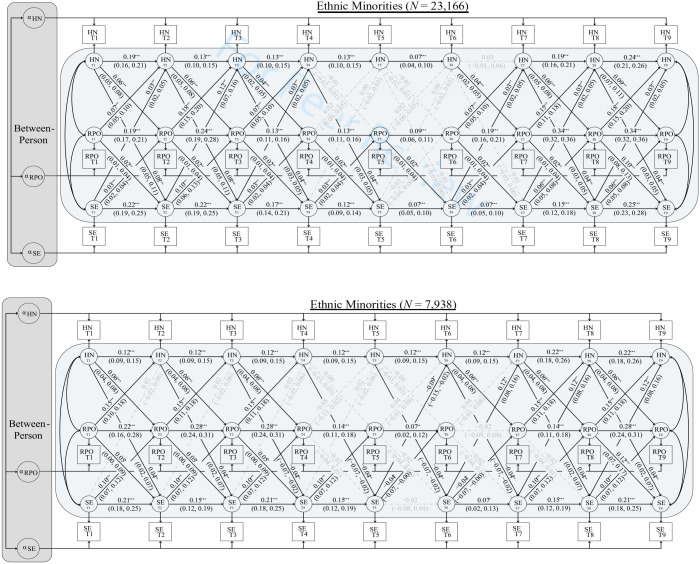
Partially Stationary Multigroup Random Intercept Cross-Lagged Panel Model of the Associations Between Historical Negation (HN), Symbolic Exclusion (SE), and Resource Policy Opposition (RPO) Among an Ethnic Majority Group (Top Panel) and Ethnic Minorities (Bottom Panel). *Note*. Our model fit these data well, χ^2^_(574)_ = 3,269.158, CFI = .992, RMSEA = .017 [.017, .018], SRMR = .049. For clarity, within-person covariances were estimated at each time point, but excluded from the figure (see Table 2). Estimates reflect unstandardised regression coefficients (with 95% confidence intervals in parentheses). ^†^*p* < .10; ^*^p < .05; ^**^p < .01; ^***^p < .001.

Turning to the within-person associations, the unstandardized regression coefficients displayed in the top panel of Figure 1 indicate that, with the exception of the autoregressive effect of historical negation at Time 6 to Time 7 (*b* = 0.03, 95% CI [−0.01, 0.06]; *p* = .111), all autoregressive effects for historical negation (*b*s = 0.07–0.24; *p*s < .001), symbolic exclusion (*b*s = 0.07–0.25; *p* < .001), and resource policy opposition (*b*s = 0.09–0.34; *p* < .001) were significant for the ethnic majority group. Whereas autoregressive effects in traditional CLPMs are interpreted as stability coefficients, they reflect inertia in RI-CLPMs. For example, a 1-unit deviation in participants’ trait level of historical negation at Time 2 carried over as a 0.13-unit deviation from their trait-level mean at Time 3.

**Table 2:** Within-Person Contemporaneous Covariances (at Time 1) and Residual Covariances (at Times 2–9) Between the Dark Duo and Resource Policy Opposition.

	Time 1	Time 2	Time 3	Time 4	Time 5	Time 6	Time 7	Time 8	Time 9
**Ethnic Majority**									
Historical Negation with Resource Policy Opposition	0.16*** (0.08, 0.14)	0.09*** (0.07, 0.10)	0.08*** (0.06, 0.09)	0.04*** (0.02, 0.05)	0.04*** (0.03, 0.05)	0.04*** (0.03, 0.05)	0.08*** (0.07, 0.09)	0.07*** (0.06, 0.08)	0.11*** (0.10, 0.12)
Symbolic Exclusion with Resource Policy Opposition	0.13*** (0.10, 0.15)	0.07*** (0.06, 0.09)	0.05*** (0.03, 0.06)	0.04*** (0.03, 0.05)	0.03*** (0.02, 0.04)	0.03*** (0.02, 0.04)	0.04*** (0.03, 0.05)	0.06*** (0.05, 0.06)	0.07*** (0.06 0.08)
Historical Negation with Symbolic Exclusion	0.11*** (0.08, 0.14)	0.09*** (0.07, 0.12)	0.07*** (0.05, 0.08)	0.05*** (0.04, 0.07)	0.04*** (0.03, 0.05)	0.01* (0.00, 0.02)	0.03*** (0.02, 0.04)	0.04*** (0.03, 0.05)	0.05*** (0.04, 0.06)
**Ethnic Minorities**									
Historical Negation with Resource Policy Opposition	0.15*** (0.10, 0.20)	0.12*** (0.08, 0.17)	0.04* (0.00, 0.08)	0.03^†^ (−0.00, 0.07)	0.02 (−0.01, 0.05)	0.02^†^ (−0.00, 0.05)	0.06*** (0.03, 0.08)	0.10*** (0.08, 0.13)	0.10*** (0.08, 0.13)
Symbolic Exclusion with Resource Policy Opposition	0.17*** (0.13, 0.22)	0.09*** (0.05, 0.14)	0.04* (0.01, 0.07)	0.09*** (0.06, 0.12)	0.05*** (0.03, 0.07)	0.03** (0.01, 0.05)	0.06*** (0.04, 0.08)	0.09*** (0.07, 0.11)	0.10*** (0.07, 0.12)
Historical Negation with Symbolic Exclusion	0.10*** (0.05, 0.15)	0.08** (0.03, 0.13)	0.01 (−0.03, 0.05)	0.04* (0.00, 0.08)	0.00 (−0.02, 0.03)	0.03^†^ (−0.00, 0.05)	0.06*** (0.03, 0.08)	0.06*** (0.03, 0.08)	0.07*** (0.04, 0.10)

*Note*. Correlations reflect unstandardized coefficients (with 95% confidence intervals in parentheses). ^†^*p* < .10; *p < .05; **p < .01; ***p < .001.

Moving to the within-person cross-lagged effects, Figure 1 reveals partial support for Hypothesis 1a. Specifically, historical negation predicted within-person increases in resource policy opposition in six out of eight cross-lagged paths. These relationships emerged after adjusting for both the autoregressive effects of resource policy opposition and the cross-lagged effects of symbolic exclusion (which were significant in seven out of eight instances). Somewhat unexpectedly, resource policy opposition also predicted within-person increases in historical negation (six out of eight times) and symbolic exclusion (all eight times). Finally, seven out of the eight cross-lagged effects of historical negation on symbolic exclusion were positive and significant, whereas six out of the eight cross-lagged effects of symbolic exclusion on historical negation were positive and significant (for the contemporaneous [residual] covariances, see Table 2).

Turning to the minority group, results again revealed positive correlations between the three random intercepts. Specifically, between-person differences in historical negation correlated positively with between-person differences in symbolic exclusion (*b* = 0.84, 95% CI [0.80, 0.89]; *p* < .001) and resource policy opposition (*b* = 1.15, 95% CI [1.10, 1.21]; *p* < .001). Between-person differences in symbolic exclusion also correlated positively with resource policy opposition (*b* = 1.13, 95% CI [1.08, 1.19]; *p* < .001). Constraining to equality the correlations the Dark Duo had with policy opposition did not significantly reduce model fit, Δ^2^_(1)_ = 0.572, *p* = .449, indicating that between-person differences in historical negation and symbolic exclusion had equally sized correlations with resource policy opposition among ethnic minorities.

The bottom panel of Figure 1 reveals the within-person associations for minorities. Excluding resource policy opposition at Time 6 to Time 7 (*b* = −0.02, 95% CI [−0.09, 0.04]; *p* = .498) and symbolic exclusion at Time 5 to Time 6 (*b* = −0.02, 95% CI [−0.08, 0.04]; *p* = .480), the autoregressive effects of historical negation (*b*s = 0.12–0.22; *p*s < .001), symbolic exclusion (*b*s = 0.07–0.21; *p*s ≤ .010), and resource policy opposition (*b*s = 0.07–0.28; *p*s ≤ .008) were all positive and significant. Likewise, six of the eight cross-lagged effects of symbolic exclusion on resource policy opposition were positive and significant (*b*s = 0.10; *p*s < .001), whereas two were *negative* and significant (*b*s = −0.04; *p*s = .043). After adjusting for these autoregressive and cross-lagged effects, we found partial support for Hypothesis 1b: Historical negation predicted within-person increases in resource policy opposition six out of eight times (*b*s = 0.06; *p*s < .001).

### Symbolic Policy Opposition

Our nonstationary multigroup RI-CLPM of the Dark Duo and symbolic policy opposition also fit these data well, χ^2^_(492)_ = 2,483.542, CFI = .994, RMSEA = .016 [.016, .017], SRMR = .032. Constraining all the congeneric paths to equality in a fully stationary model did, however, significantly reduce model fit, χ^2^_(618)_ = 3,251.996, CFI = .992, RMSEA = .017 [.016, .017], SRMR = .034; Δ^2^_(126)_ = 768.472, *p* < .001. As such, we estimated a partial stationary model by constraining to equality all the congeneric paths that did not significantly reduce model fit relative to the non-stationary model, χ^2^_(577)_ = 2,533.978, CFI = .994, RMSEA = .015 [.014, .015], SRMR = .032; Δ^2^_(85)_ = 50.436, *p* = .999. Because this model is more parsimonious than the nonstationary model and does not compromise model fit, we report the unstandardized regression coefficients for our partially stationary model below.

Focusing first on the between-person components of the model for the ethnic majority group, results revealed that the three random intercepts correlated positively with each other. Specifically, between-person differences in symbolic exclusion correlated positively with both historical negation (*b* = 1.05, 95% CI [1.02, 1.08]; *p* < .001) and symbolic policy opposition (*b* = 1.36, 95% CI [1.33, 1.39]; *p* < .001). Historical negation also correlated positively with symbolic policy opposition (*b* = 0.77, 95% CI [0.74, 0.79]; *p* < .001). Constraining to equality the correlations the Dark Duo had with policy opposition did, however, reduce model fit, Δ^2^_(1)_ = 3164.026, *p* < .001. Thus, between-person differences in symbolic exclusion correlated more strongly with symbolic policy opposition than did historical negation among the ethnic majority group.

**Figure 2:**
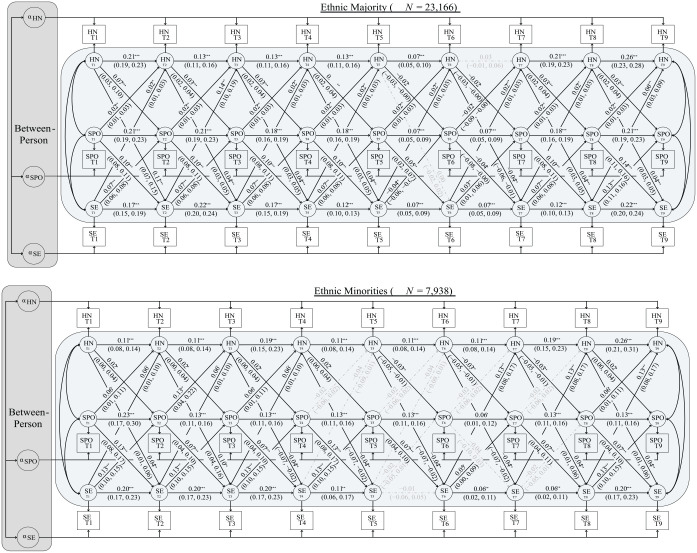
Partially Stationary Multigroup Random Intercept Cross-Lagged Panel Model of the Associations Between Historical Negation (HN), Symbolic Exclusion (SE), and Symbolic Policy Opposition (SPO) Among an Ethnic Majority Group (Top Panel) and Ethnic Minorities (Bottom Panel). *Note*. Our model fit these data well, χ^2^_(577)_ = 2,533.978, CFI = .994, RMSEA = .015 [.014, .015], SRMR = .032. For clarity, within-person covariances were estimated at each time point, but excluded from the figure (see Table 3). Estimates reflect unstandardised regression coefficients (with 95% confidence intervals in parentheses). *p < .05; **p < .01; ***p < .001.

The top panel of Figure 2 displays the within-person associations among the ethnic majority group. Once again excluding the nonsignificant autoregressive effect of historical negation at Time 6 to Time 7 (*b* = 0.03, 95% CI [−0.01, 0.06]; *p* = .119), all autoregressive effects for historical negation (*b*s = 0.07 to 0.26; *p*s < .001), symbolic exclusion (*b*s = 0.07 to 0.22; *p* < .001), and symbolic policy opposition (*b*s = 0.07 to 0.21; *p* < .001) were significant. And consistent with Hypothesis 2a, within-person departures from participants’ trait-level symbolic exclusion predicted within-person increases in symbolic policy opposition seven out of eight times. Unexpectedly, the cross-lagged effect of symbolic exclusion at Time 5 on symbolic policy opposition at Time 6 was negative (*b* = −0.04, 95% CI [−0.06, −0.01]; *p* = .002). These relationships emerged after adjusting for both the autoregressive effects of symbolic policy opposition, and the cross-lagged effects of historical negation (which were significant and positive six out of eight times). Symbolic policy opposition also predicted within-person increases in both symbolic exclusion and historical negation in seven out of eight time points (see Table 3 for the contemporaneous [residual] covariances).

**Table 3.** Within-Person Correlations Between Contemporaneous Covariances (at Time 1) and Residual Covariances (at Times 2-9) Between the Dark Duo and Symbolic Policy Opposition.

	Time 1	Time 2	Time 3	Time 4	Time 5	Time 6	Time 7	Time 8	Time 9
Ethnic Majority									
Historical Negation with Symbolic Policy Opposition	0.03** (0.01, 0.06)	0.03** (0.01, 0.05)	0.02* (0.00, 0.03)	0.02* (0.00, 0.03)	0.01^†^ (−0.00, 0.02)	−0.01^†^ (−0.02, 0.00)	−0.00 (−0.01, 0.01)	0.02*** (0.01, 0.03)	0.02*** (0.01, 0.03)
Symbolic Exclusion with Symbolic Policy Opposition	0.28*** (0.25, 0.31)	0.16*** (0.14, 0.18)	0.13*** (0.12, 0.15)	0.11*** (0.10, 0.12)	0.10*** (0.09, 0.11)	0.06*** (0.05, 0.07)	0.08*** (0.07, 0.09)	0.11*** (0.10, 0.12)	0.15*** (.014, 0.16)
Historical Negation with Symbolic Exclusion	0.11*** (0.08, 0.14)	0.10*** (0.07, 0.12)	0.07*** (0.05, 0.08)	0.06*** (0.04, 0.07)	0.05*** (0.04, 0.06)	0.01 (−0.00, 0.02)	0.01* (0.00, 0.03)	0.04*** (0.03, 0.05)	0.05*** (0.04, 0.06)
Ethnic Minorities									
Historical Negation with Symbolic Policy Opposition	0.07** (0.02, 0.11)	0.06** (0.02, 0.11)	−0.01 (−0.04, 0.03)	−0.01 (−0.04, 0.02)	−0.01 (−0.04, 0.01)	−0.02* (−0.05, −0.00)	−0.01 (−0.03, 0.01)	−0.01 (−0.03, 0.01)	0.03* (0.00, 0.05)
Symbolic Exclusion with Symbolic Policy Opposition	0.28*** (00.23, 0.32)	0.18*** (0.14, 0.22)	0.13*** (0.10, 0.16)	0.17*** (0.15, 0.20)	0.11*** (0.09, 0.13)	0.06*** (0.04, 0.08)	0.07*** (0.05, 0.09)	0.09*** (0.07, 0.11)	0.14*** (0.12, 0.16)
Historical Negation with Symbolic Exclusion	0.11*** (0.05, 0.16)	0.09*** (0.04, 0.14)	0.02 (−0.02, 0.06)	0.04* (0.01, 0.08)	0.01 (−0.01, 0.04)	0.03^†^ (−0.00, 0.05)	0.05*** (0.03, 0.08)	0.04** (0.02, 0.07)	0.07*** (0.05, 0.10)

*Note*. Correlations reflect unstandardized coefficients (with 95% confidence intervals in parentheses). ^†^*p* < .10; *p < .05; **p < .01; ***p < .001.

As for the minority group, positive correlations between the three random intercepts were again present. Specifically, between-person differences in symbolic exclusion correlated positively with between-person differences in historical negation (*b* = 0.84, 95% CI [0.80, 0.89]; *p* < .001) and symbolic policy opposition (*b* = 0.92, 95% CI [0.89, 0.95]; *p* < .001). Between-person differences in historical negation also correlated positively with symbolic policy opposition (*b* = 0.50, 95% CI [0.46, 0.54]; *p* < .001). Constraining to equality the correlations the Dark Duo had with symbolic opposition significantly reduced model fit, Δ^2^_(1)_ = 574.771, *p* < .001. Thus, between-person differences in symbolic exclusion correlated more strongly with symbolic policy opposition than did historical negation among ethnic minorities.

The within-person associations for minorities appear in the bottom panel of Figure 2. Except for symbolic exclusion at Time 5 to Time 6 (*b* = −0.01, 95% CI [−0.06, 0.05]; *p* = .830), all the autoregressive effects for symbolic exclusion (*b*s = 0.06 to 0.20; *p*s ≤ .008), historical negation (*b*s = 0.11–0.26; *p*s < .001), and symbolic policy opposition (*b*s = 0.06–0.23; *p*s ≤ .028) were positive and significant. As for the cross-lagged effects, deviations from one’s trait-level historical negative predicted within-person increases in symbolic policy opposition five out of eight times (*b*s = 0.02; *p*s = .033), and within-person decreases in symbolic policy opposition the remaining three times (*b*s = −0.03, 95% CI [−0.05, −0.01]; *p* = .001). After adjusting for these autoregressive and cross-lagged effects, we found support for Hypothesis 2b: Symbolic exclusion predicted within-person increases in symbolic policy opposition six out of eight times (*b*s = 0.05–13; *p*s ≤ .035). Unexpectedly, the remaining two cross-lagged associations between symbolic exclusion and symbolic policy opposition were nonsignificant (*b*s = −0.02; *p*s = .192).

## Discussion

Over the past decade, the Dark Duo literature has examined how two culture-specific ideologies maintain colonial inequities in settler colonial settings. To date, several political routes through which historical negation and symbolic exclusion inhibit racial equality have been identified. For example, the Dark Duo undermines support for both progressive political parties (Greaves et al., 2014; Sibley, 2010; Sibley & Osborne, 2016) and Indigenous-oriented collective action (Osborne et al., 2017). Importantly, the Dark Duo are also associated with opposition to bicultural policy (Newton et al., 2018; Satherley & Sibley, 2018; Sibley & Liu, 2012; Sibley et al., 2008). However, the temporal ordering of the relationship between symbolic exclusion and support for both resource-based and symbolic bicultural policies is yet to be examined. Likewise, research must clarify the direction of the relationship between historical negation and symbolic policy attitudes, particularly given that past longitudinal research has confounded between-person stability with within-person change. Given the critical role that specific bicultural policies can play in redressing contemporary inequities, understanding the direct ways in which ideology influences—and is influenced by—policy support is crucial.

### Core Findings and Implications

This study addresses previous limitations in the literature by using innovative statistical modeling to reveal the temporal ordering of within-person changes in endorsement of the Dark Duo and bicultural policy support over time. Consistent with Hypotheses 1a and 1b, departures from one’s trait-level historical negation preceded increases in opposition to resource-based bicultural policies 1 year later in six of the eight cross-lagged paths examined. Notably, these results held for both the majority and minority ethnic groups. Thus, the perception that past injustices toward the Indigenous group are irrelevant to contemporary inequalities undermines subsequent support for policies that promote resource-based equality.

Also as hypothesized, symbolic exclusion had a positive cross-lagged effect on opposition to symbolic-based bicultural policies 1 year later for both the majority (Hypothesis 2a) and the minority (Hypothesis 2b) ethnic groups. These results replicated across seven of the eight cross-lagged paths for the ethnic majority group, and six of the eight cross-lagged paths for the ethnic minority group. Thus, the belief that Indigenous culture is irrelevant to the superordinate national identity generally undermines support for policies that aim to incorporate aspects of this culture into mainstream society.

Collectively, our results support the Dark Duo Model’s assertion that historical negation and symbolic exclusion function together to maintain inequality between the settler colonizer group and the Indigenous group in settler colonial nations (Sibley, 2010). These results complement and extend past work on the political implications of the Dark Duo by demonstrating that historical negation and symbolic exclusion promote political choices that obstruct social change toward racial equality (see Greaves et al., 2014; Osborne et al., 2017; Sibley, 2010; Sibley et al., 2008; Sibley & Liu, 2012; Sibley & Osborne, 2016). Given the enduring systematic change bicultural policies evoke (above and beyond party votes and protest), our results extend this literature to aid a more comprehensive understanding of how the Dark Duo sustains the colonial project both ideologically *and* politically.

To achieve equality within a socio-historical context of colonization, the cascade of intergenerational inequality must be disrupted and repaired at a systemic level (e.g., through political intervention). Historical negation and symbolic exclusion support the succession of harm by impeding specific policies that make targeted reparations for colonial injustices and incorporate Indigenous culture into mainstream systems. By illustrating the harmful effects of these ideologies, our results elucidate the Dark Duo’s ability to extend the colonial project by retaining the privilege and social status of the settler colonizer group at the expense of Indigenous peoples. Although our work thus illustrates the dire effects of the Dark Duo, it also identifies historical negation and symbolic exclusion as potential targets for interventions that challenge colonial inequality.

This study provides important insights into how culture-specific ideologies maintain social inequities. In the same vein as other contemporary forms of racism (see Dovidio et al., 2016), the Dark Duo is tailored to the unique conditions of the socio-historical context and maintains the current hierarchy in a way that resonates with that culture (Sibley, 2010). Specifically, historical negation and symbolic exclusion attend to the indisputable past injustices toward Indigenous peoples and their undeniable nationality by disregarding the relevance of these factors to legitimize racial inequalities. Our results thus illustrate how these legitimizing myths produce social outcomes through political processes (namely, opposition to specific policies). Although the Dark Duo literature predominantly examines these ideologies in Aotearoa, historical negation and symbolic exclusion also operate in (or in relation to) other colonization contexts including Chile (Castro et al., 2022) and Puerto Rico (Rivera Pichardo et al., 2022). Nevertheless, as socio-structural conditions differ between cultures and evolve over time, it is critical to understand the powerful ideologies that are tailored to unique national contexts.

### Bidirectional Pathways

Our examination of the reciprocal associations between the Dark Duo and bicultural policy opposition revealed that opposition to resource- and symbolic-based policies generally predicted increases in historical negation and symbolic exclusion over time, respectively. In fact, these cross-lagged effects were roughly the same size (or larger) than the corresponding cross-lagged effects of the Dark Duo on policy support for both the majority and the minority ethnic groups. Thus, although within-person increases in historical negation and symbolic exclusion precede subsequent increases in opposition to resource- and symbolic-based policies, bicultural policy opposition also reinforces these ideologies. These results corroborate Greaves and colleagues’ (2014) discovery that the Dark Duo both promote and are promoted by conservative party preferences. Our results also help to clarify conflicting evidence from past research regarding the influence of symbolic policy attitudes on historical negation (Sibley & Liu, 2012).

Although unidirectional paths are often favored in the social sciences for their parsimony, bidirectional relationships are consistent with the dynamicity and complexity of human behavior. Indeed, capturing the reciprocal effects prevalent among psychological processes (including political attitudes; e.g., Greaves et al., 2014; Satherley et al., 2023) is crucial for an accurate understanding of the human experience and allows for more nuanced and effective interventions. Here, the bidirectionality of these associations indicates that the endorsement of historical negation and symbolic exclusion could trigger a self-perpetuating cycle of anti-biculturalism between ideology and policy preferences. This is an important discovery that demonstrates the self-maintenance of the Dark Duo and policy opposition in settler colonial contexts. However, it also reveals two points of interception for disrupting this colonizing cycle and promoting progressive action.

Collectively, our results contribute to the broader understanding of the relationship between ideologies and policy support by showing how societal inequities are maintained through both top-down and bottom-up processes. Specifically, abstract ideologies influence policy support, while specific policy preferences synchronously influence ideology. This bottom-up path may reflect postbehavior rationalization, whereby individuals change their beliefs to justify their behaviors (Festinger, 1957; also see Beasley & Joslyn, 2001). Yet this study also demonstrates the power of ideology in determining social outcomes. These results corroborate an expansive literature showing that broader belief systems including conservatism and other system-justifying ideologies operate through general political preferences to inform social outcomes (see Jost et al., 2003; Jost & Hunyady, 2005). Thus, our results endorse Jost’s (2006) assertion that ideology is alive and well in the public (see also Gries et al., 2022; but see Osborne et al., 2022). Although this approach can have unjust consequences in the case of ideologies that foster inequality, it also allows social structures to evolve with changing beliefs and suggests that equality-oriented belief systems could propel progressive social change.

### Spill-Over Effects and the Nuances of Ethnic Group Membership

In addition to obtaining support for our hypothesized relationships, we found significant spill-over effects for both the majority and the minority ethnic groups. Specifically, symbolic exclusion generally predicted increases in opposition to resource-based bicultural policies over time (for both the majority and the minority groups). Moreover, the strength of these cross-lagged effects was often comparable to the cross-lagged effects of historical negation on resource policy opposition. Conversely, although historical negation typically predicted increases in opposition to symbolic-based bicultural policies over time (for the majority group), symbolic exclusion was clearly the stronger predictor. Given the Dark Duo are correlated, but distinct, ideologies that mutually justify the status quo (Sibley, 2010), it is unsurprising that they have common political implications. However, our results demonstrate that historical negation is more distinctly tied to a resource-based agenda, whereas symbolic exclusion propels a broader colonial goal. These novel results place symbolic exclusion as a particularly meaningful target for interventions to enhance political support for biculturalism.

The replication of our results across the ethnic majority group (the settler colonizers) and a group of ethnic minorities (inclusive of Māori) demonstrates the powerful antibicultural effects of the Dark Duo. Indeed, the bidirectional associations between the Dark Duo and bicultural policy opposition reflect a vicious cycle of legitimization whereby ideologies that justify colonial inequities undermine support for redistributive policies which, in turn, reinforce those same harmful ideologies. While the legitimizing effects emerge across groups, their underlying motivations likely differ (e.g., see Bahamondes et al., 2022). Indeed, this cycle accords with the self-interest of the majority group and protects their self-image by justifying their privilege (see Jost & Hunyady, 2003). Conversely, legitimizing inequities undermines the material interests of the disadvantaged group, but may offer a palliative reprieve from the emotional weight of recognizing this injustice (see Jost & Hunyady, 2003). Thus, in the face of an unfair and seemingly unchangeable system, this legitimization may present as the only adaptive path forward for groups confronted with deeply embedded colonial inequalities.

### Limitations, Strengths, and Future Directions

There are several limitations to our study. First, one of our booster samples recruited participants via the website of a nationwide newspaper. Although this group comprised only 9.5% of our overall sample, these self-selection biases reduce the generalizability of our results. Analyses excluding this nonrandom subsample did, however, yield substantively identical results (see Figures S1a–S2b in the Online Supplement). Second, despite being a (mostly) random sample of the adult population, our sample over-represented participants who identified as female. Our results do, however, generally replicate across nine annual waves of data. Such an extensive assessment period enables a particularly reliable estimate of the year-to-year effects of ideology on policy support, as well as the between-person trait-like components to these separate constructs. Thus, although our sample does not represent Aotearoa perfectly, our large sample size and extended temporal focus provides an unprecedented examination of the policy implications of the Dark Duo and increases confidence in our conclusions.

Although our sample size was large, our multigroup analyses required us to examine minorities as a pan-ethnic group. Ideally, we would focus exclusively on Māori, as the Dark Duo is specific to relations between the settler colonizers and the Indigenous peoples of Aotearoa. Ethnic minorities (i.e., Māori, Pacific Islanders, and Asians) do, however, experience elevated rates of microaggressions and discrimination across Aotearoa relative to Pākehā (see Harris et al., 2006; Harris et al., 2018). Thus, other ethnic minorities share with Māori a common experience of marginalization in Aotearoa. At a broader level, the colonial agenda propels Western self-interest at the detriment of “other” cultures. Accordingly, the system-justifying processes within these groups may align more with the Indigenous peoples than with the settler colonizers. Nonetheless, future research is needed to examine these processes separately for each ethnic minority group.

Related to the sample size requirements for our multigroup analyses, it is important to discuss our effect sizes. Because RI-CLPMs partition participants’ responses into within-person and between-person processes, they generally yield small effect sizes (see Orth et al., in press; Osborne & Little, in press). Indeed, we identified small cross-lagged effects between the Dark Duo and policy attitudes. However, as Götz and colleagues (2022) note, small effect sizes should be the norm in psychological science given the multiply determined nature of complex human behavior. Consistent with this view, a recent meta-analysis of cross-lagged models indicated that most RI-CLPMs yield effect sizes that range between .02 and .11 (Orth et al., in press)—a range that captures the effects reported in this study. Nevertheless, these small effect sizes mean that other factors impact policy support, and vice versa. Future research should focus on uncovering some of these factors.

Although traditional CLPMs have typically been used to reveal the temporal ordering of constructs, this approach confounds trait-like stability and within-person change (see Berry & Willoughby, 2017; Hamaker et al., 2015; Osborne & Little, in press). As such, our use of RI-CLPMs allows us to focus on the within-person processes that are central to psychological theory by adjusting for the trait-like stability of both the Dark Duo and policy support via the estimation of random intercepts. Accordingly, our results are the first to demonstrate that within-person changes in historical negation and symbolic exclusion *precede* within-person changes in opposition to bicultural policies, as well as vice versa (i.e., a bidirectional effect). Moreover, these results replicated across most of the annual waves of the study for both the ethnic majority and ethnic minority groups. As such, this study provides the strongest evidence to date that the Dark Duo temporally precedes people’s bicultural policy attitudes.

By affirming the political power of the Dark Duo in maintaining social inequalities between the settler colonizers and the Indigenous peoples, this study helps pave the way for social change strategies in settler colonial contexts. Indeed, our results identify historical negation and symbolic exclusion as valuable points of interception for disrupting the perpetuation of racial inequality. Thus, future research exploring ways to undermine the Dark Duo could aid progressive social change. Given the Dark Duo deny the relevance of past injustices and the Indigenous culture to contemporary Aotearoa, consciousness-raising on (a) how colonization creates current inequities and (b) the value of Indigenous representation may be strategies worth exploring. For the Indigenous group,^4^ this could tackle false consciousness promoted by system-justifying ideologies (Jost & Hunyady, 2003). Conversely, education on the history of Māori and colonization may foster progressive attitudes and beliefs surrounding racial equality among the settler colonizer group (see Pack et al., 2016).

## Conclusion

As social change movements seek to rectify the ongoing harm of colonization on Indigenous communities, research examining the processes that aid or impede redressing the enduring inequities between the settler colonizers and Indigenous peoples is ever more important. To these ends, the Dark Duo literature identifies two distinct ideologies tailored to the settler colonial context that legitimize current inequalities. Expanding previous research on the political implications of historical negation and symbolic exclusion, this study examined the temporal ordering of the relationships between the Dark Duo and support for resource-based and symbolic-based bicultural policies. As expected, historical negation and symbolic exclusion predicted within-person increases in opposition to resource-based and symbolic-based bicultural policies (respectively) over time. Notably, these associations held for both the majority and minority ethnic groups of Aotearoa, replicated across most of the nine annual waves assessed here, and were generally bidirectional. This study thus identifies a self-perpetuating cycle between colonial ideologies and the policies that maintain—and exacerbate—inequities in settler colonial contexts.

## Supplemental Material

sj-docx-1-psp-10.1177_01461672231209657 – Supplemental material for Barriers to Biculturalism: Historical Negation and Symbolic Exclusion Predict Longitudinal Increases in Bicultural Policy Opposition Supplemental material, sj-docx-1-psp-10.1177_01461672231209657 for Barriers to Biculturalism: Historical Negation and Symbolic Exclusion Predict Longitudinal Increases in Bicultural Policy Opposition by Zoe Bertenshaw, Chris G. Sibley and Danny Osborne in Personality and Social Psychology Bulletin

## Notes

1. Though the Dark Duo has been described as a model of *post-colonial* ideology, we refer to it as a model of *colonial* ideology to recognize the enduring nature of colonization.

2. Due to convergence issues involving the Time 7 measures of symbolic exclusion and symbolic policy opposition, we examined the measurement invariance of the Dark Duo separately from policy opposition.

3. See Footnote 1.

4. Such interventions should, however, be led by Indigenous peoples and are likely to be most effective when drawing on Indigenous knowledge and practices (González et al., 2022).
