# Supplementary material for: Barriers to Biculturalism: Historical Negation and Symbolic Exclusion Predict Longitudinal Increases in Bicultural Policy Opposition
Source: Pers Soc Psychol Bull. 2023 Nov 9;51(6):967–83. doi: 10.1177/01461672231209657 (PMC12044213; doi:10.1177/01461672231209657)
Supplement: sj-docx-1-psp-10.1177_01461672231209657 – Supplemental material for Barriers to Biculturalism: Historical Negation and Symbolic Exclusion Predict Longitudinal Increases in Bicultural Policy Opposition [file sj-docx-1-psp-10.1177_01461672231209657.docx]

**Online Supplement**

Our data come from an ongoing longitudinal study (namely, the New Zealand Attitudes and Values Study) that began in 2009. Although most of our participants have been randomly selected from the New Zealand electoral roll (i.e., a mandatory registry of eligible voters^[[1]](#footnote-1)^), a small percentage (i.e., 9.5%) volunteered to participate via an online newspaper in 2011. Because the non-random nature of their entry into the sample frame could introduce self-selection biases into our study and compromise the generalisability of our results, we re-ran our focal multi-group random intercept cross-lagged panel models. Consistent with the approach employed in our manuscript, we estimated our models with partial stationarity and handled missing data using full information maximum likelihood estimates.

The results of our supplementary analyses excluding participants who self-selected into the study via the online newspaper are displayed in Figures S1 (for the Dark Duo and resource policy opposition) and S2 (for the Dark Duo and symbolic policy opposition). As shown here, the results for resource policy opposition were virtually unchanged for both the ethnic majority group (top panel) and ethnic minorities (bottom panel). The few changes that did occur were the non-focal reciprocal associations between resource policy opposition and symbolic exclusion for minorities. Specifically, the cross-lagged effects of resource policy opposition on symbolic exclusion changed from being statistically significant to marginally significant at three time points, and the two unexpected negative cross-lagged effects of symbolic exclusion on resource policy opposition that emerged in our main analyses became non-significant in our supplemental analyses. That said, none of the results testing Hypothesis 1a or 1b changed when excluding the subsample who entered the study via the online newspaper.

**Figure S1.**

*Partially stationary multi-group random intercepts cross-lagged panel model of the associations between historical negation (HN), symbolic exclusion (SE), and resource policy opposition (RPO) amongst an ethnic majority group (top panel) and ethnic minorities (bottom panel).*


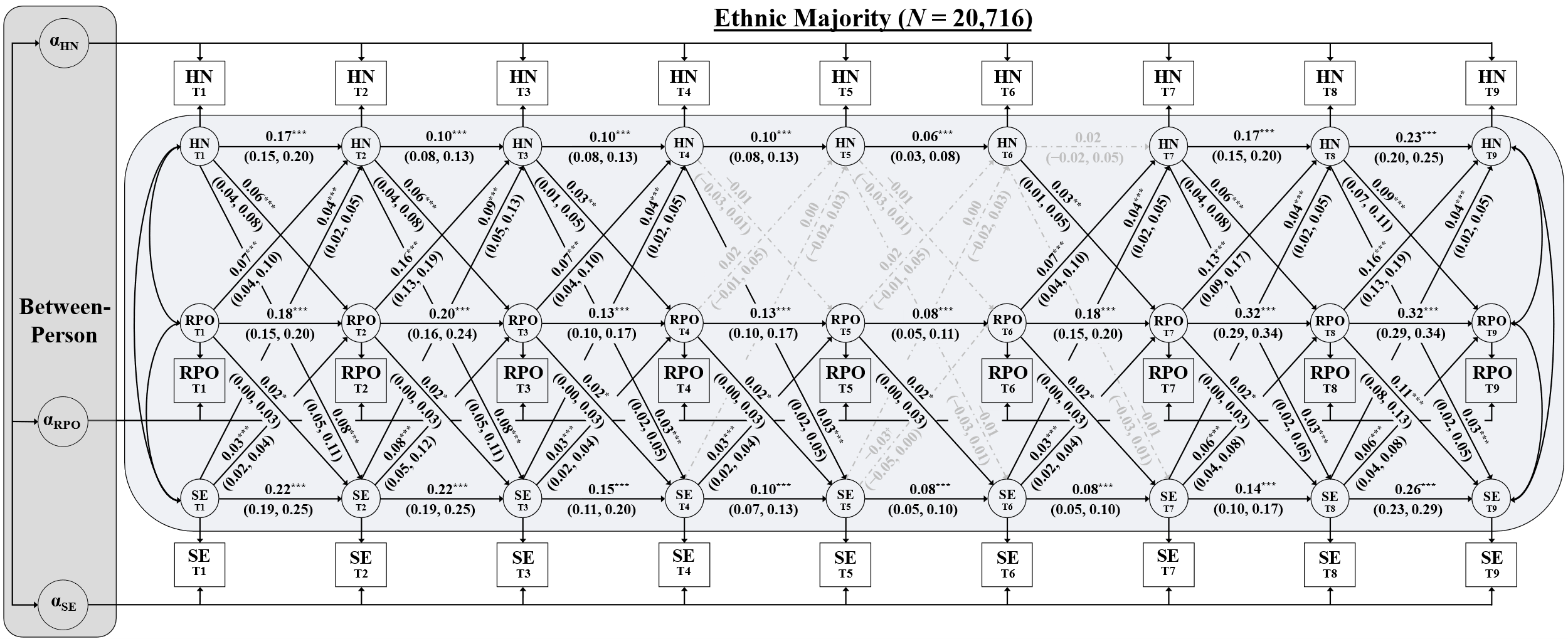


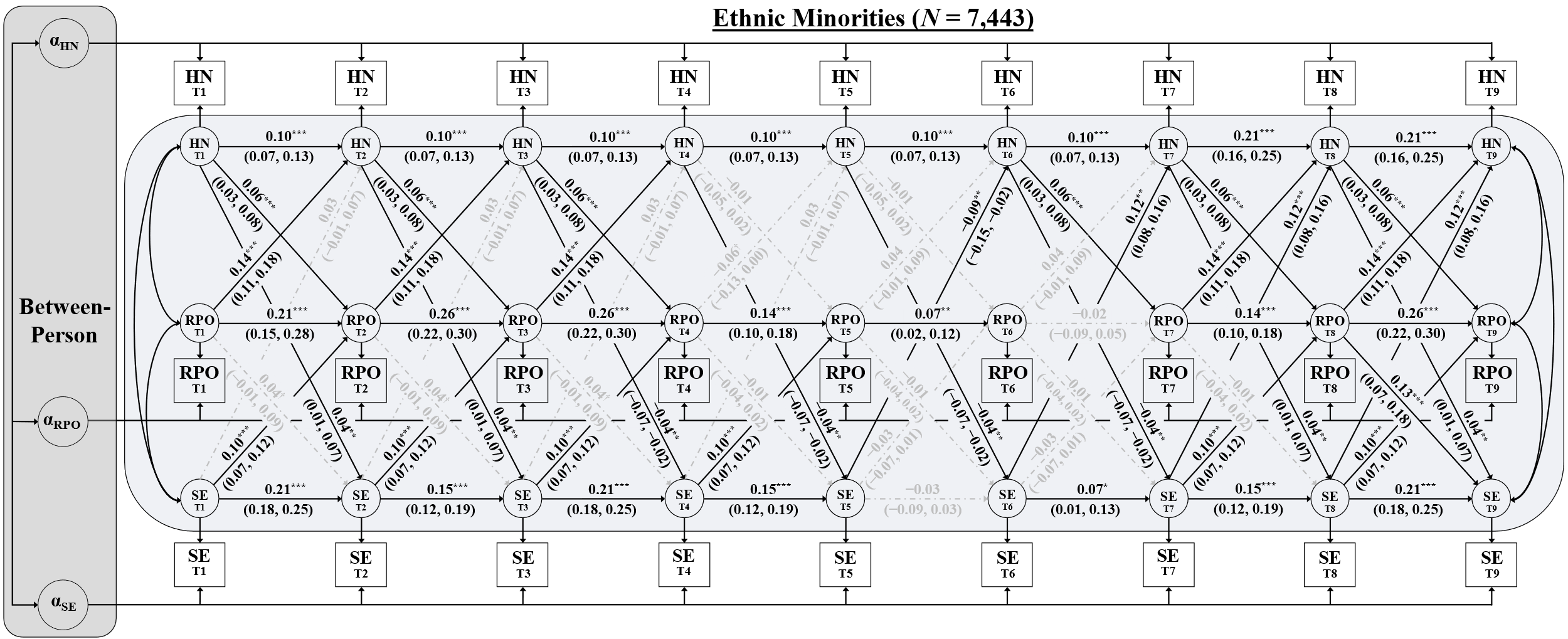


*Note*. Our model fit these data well, χ^2^ _(574)_ = 2697.719, CFI = .993, RMSEA = .016 [.016, .017], SRMR = .047. For clarity, within-person covariances were estimated at each time point, but excluded from the figure. Estimates reflect unstandardised regression coefficients (with 95% confidence intervals in parentheses). ^†^*p* < .10; ^*^*p* < .05; ^**^*p* < .01; ^***^*p* < .001.

Figure S2 displays the results of our supplemental analyses examining the Dark Duo and symbolic policy opposition. These results once again illustrate that excluding participants who self-selected into the study at Time 3 through the online newspaper did not substantively change our results. Indeed, although two of the non-hypothesized negative cross-lagged effects of historical negation on symbolic policy opposition became non-significant amongst the majority group after excluding these non-randomly sampled participants, none of the results testing Hypothesis 2a changed in these supplemental analyses. As for the results for the minority group, five of the non-hypothesized cross-lagged effects of historical negation on symbolic policy opposition became non-significant after excluding participants who self-selected into the study at Time 3. But most critically, only one of the hypothesized positive cross-lagged effects of symbolic exclusion on symbolic policy opposition changed from being significant to marginally significant in these supplemental analyses. In other words, five of the six positive cross-lagged effects supporting Hypothesis 2b remained significant when excluding participants who self-selected into the study at Time 3. Together, these supplemental analyses demonstrate that our results do not substantively change if we exclude participants who self-selected into the study at Time 3.

**Figure S2**

*Partially stationary multi-group random intercepts cross-lagged panel model of the associations between historical negation (HN), symbolic exclusion (SE), and symbolic policy opposition (SPO) amongst an ethnic majority group (top panel) and ethnic minorities (bottom panel).*


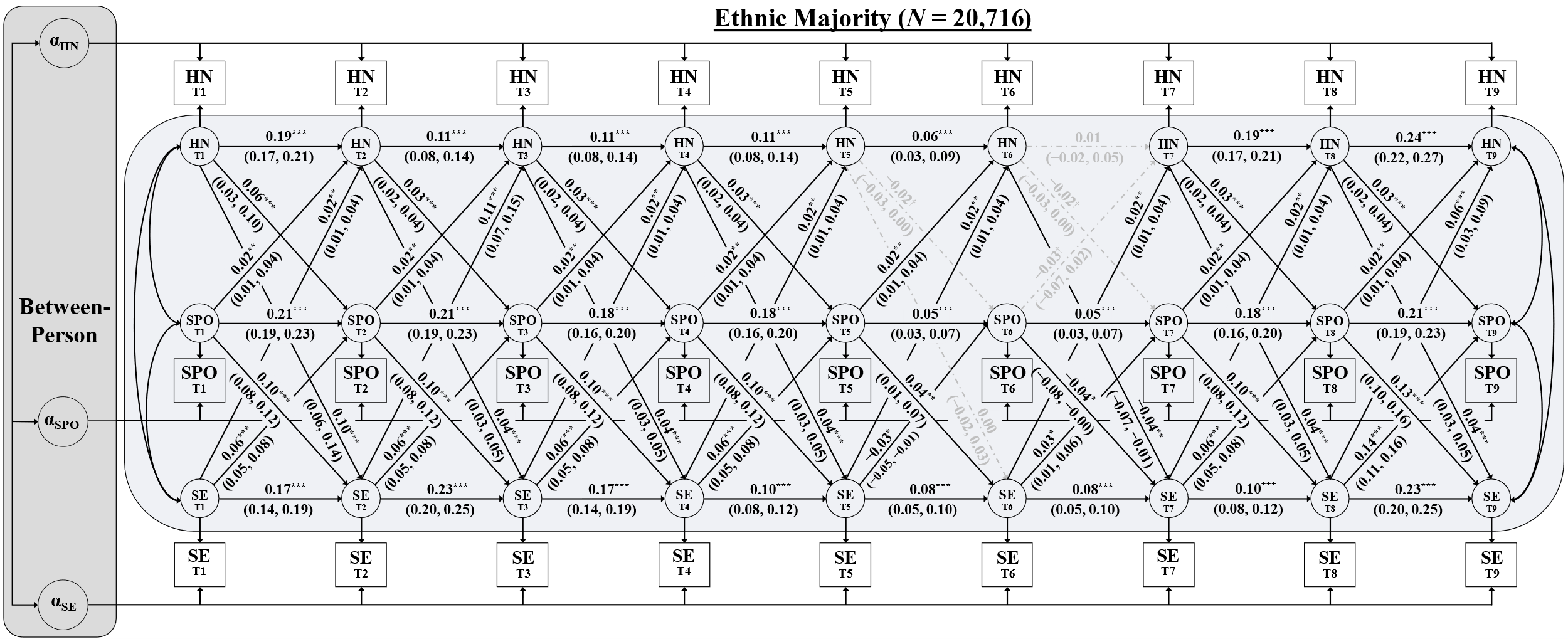


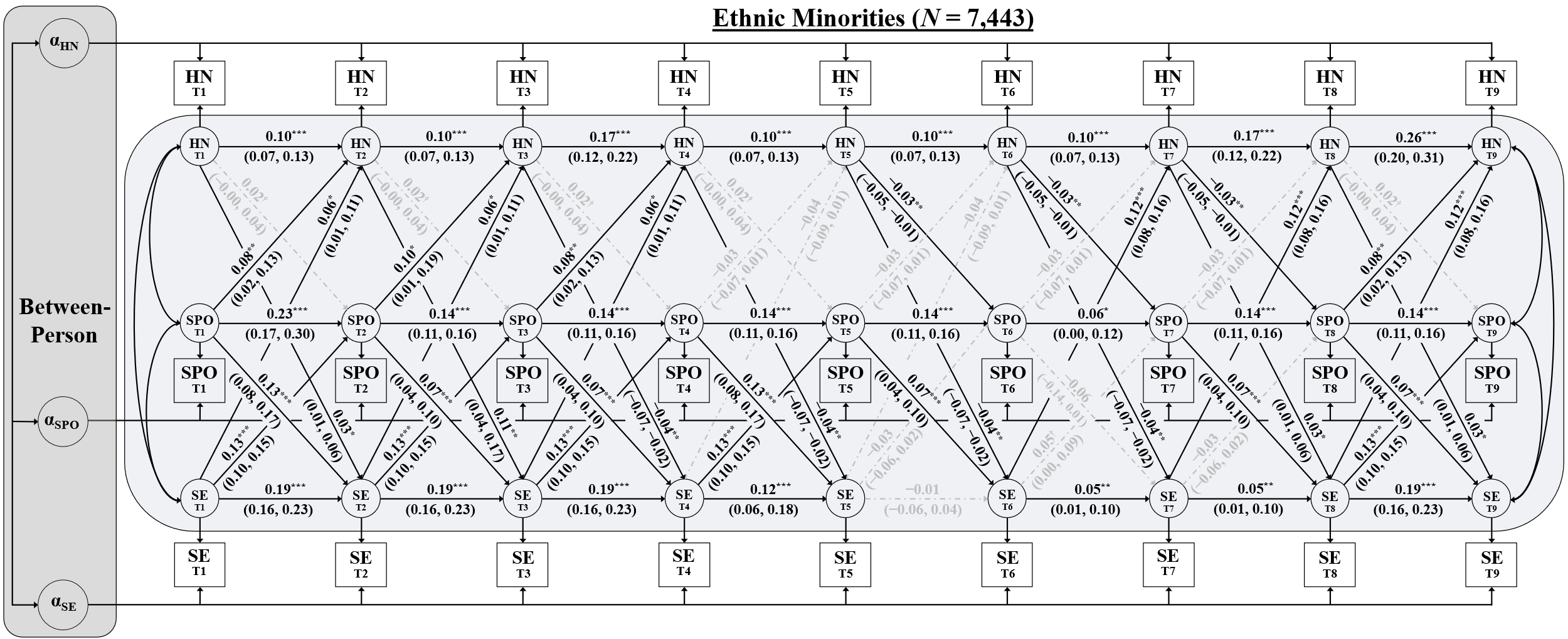


*Note*. Our model fit these data well, χ^2^ _(577)_ = 2214.411, CFI = .994, RMSEA = .014 [.014, .015], SRMR = .031. For clarity, within-person covariances were estimated at each time point, but excluded from the figure. Estimates reflect unstandardised regression coefficients (with 95% confidence intervals in parentheses). ^†^*p* < .10; ^*^*p* < .05; ^**^*p* < .01; ^***^*p* < .001.

**Descriptive Statistics and Bivariate Correlations**

Readers may be interested in the descriptive statistics and bivariate correlations between the variables presented in our manuscript. Accordingly, Table S1 presents this information for the majority group, and Table S2 presents this information for the ethnic minorities in our sample.

Table S1. *Descriptive statistics and bivariate correlations for the variables included in this study (ethnic majority group).*

|  |  | Mean | SD | α | 1 | 2 | 3 | 4 | 5 | 6 | 7 | 8 | 9 | 10 | 11 |
| --- | --- | --- | --- | --- | --- | --- | --- | --- | --- | --- | --- | --- | --- | --- | --- |
| 1. Gender^1^ |  | 0.39 | 0.49 | --- | -- |  |  |  |  |  |  |  |  |  |  |
| 1. Age^2^ |  | 43.34 | 14.59 | --- | .09 | -- |  |  |  |  |  |  |  |  |  |
| 1. Historical Negation_T1_ |  | 5.38 | 1.38 | .80 | .05^**^ | .14 | -- |  |  |  |  |  |  |  |  |
| 1. Historical Negation_T2_ |  | 5.36 | 1.36 | .80 | .06^**^ | .15 | .74 | -- |  |  |  |  |  |  |  |
| 1. Historical Negation_T3_ |  | 5.12 | 1.46 | .84 | .09 | .17 | .74 | .75 | -- |  |  |  |  |  |  |
| 1. Historical Negation_T4_ |  | 5.10 | 1.48 | .84 | .08 | .14 | .72 | .72 | .78 | -- |  |  |  |  |  |
| 1. Historical Negation_T5_ |  | 5.05 | 1.47 | .84 | .07 | .10 | .70 | .73 | .76 | .79 | -- |  |  |  |  |
| 1. Historical Negation_T6_ |  | 4.98 | 1.47 | .84 | .09 | .14 | .70 | .73 | .77 | .77 | .78 | -- |  |  |  |
| 1. Historical Negation_T7_ |  | 4.97 | 1.50 | .83 | .11 | .15 | .69 | .72 | .74 | .75 | .75 | .78 | -- |  |  |
| 1. Historical Negation_T8_ |  | 4.90 | 1.56 | .85 | .10 | .12 | .69 | .71 | .74 | .76 | .76 | .79 | .81 | -- |  |
| 1. Historical Negation_T9_ |  | 4.78 | 1.61 | .85 | .10 | .13 | .67 | .69 | .72 | .74 | .74 | .77 | .80 | .80 | -- |
| 1. Symbolic Exclusion_T1_ |  | 3.41 | 1.57 | .83 | .16 | .12 | .45 | .43 | .45 | .45 | .44 | .43 | .44 | .46 | .44 |
| 1. Symbolic Exclusion_T2_ |  | 3.41 | 1.57 | .84 | .18 | .18 | .47 | .48 | .49 | .48 | .47 | .46 | .46 | .46 | .47 |
| 1. Symbolic Exclusion_T3_ |  | 3.05 | 1.50 | .84 | .20 | .21 | .45 | .45 | .52 | .52 | .52 | .51 | .51 | .53 | .52 |
| 1. Symbolic Exclusion_T4_ |  | 3.18 | 1.55 | .86 | .21 | .20 | .46 | .45 | .53 | .53 | .54 | .52 | .54 | .55 | .55 |
| 1. Symbolic Exclusion_T5_ |  | 3.00 | 1.48 | .85 | .18 | .14 | .45 | .44 | .51 | .52 | .51 | .50 | .51 | .54 | .54 |
| 1. Symbolic Exclusion_T6_ |  | 2.89 | 1.48 | .85 | .18 | .15 | .43 | .44 | .50 | .51 | .50 | .50 | .51 | .53 | .55 |
| 1. Symbolic Exclusion_T7_ |  | 2.80 | 1.46 | .85 | .18 | .18 | .43 | .44 | .50 | .49 | .49 | .49 | .50 | .52 | .53 |
| 1. Symbolic Exclusion_T8_ |  | 2.72 | 1.43 | .85 | .16 | .15 | .41 | .42 | .49 | .49 | .48 | .48 | .50 | .51 | .52 |
| 1. Symbolic Exclusion_T9_ |  | 2.64 | 1.47 | .87 | .17 | .17 | .43 | .44 | .50 | .50 | .48 | .48 | .51 | .51 | .53 |
| 1. Resource Policy_T1_ |  | 5.61 | 1.27 | .77 | .13 | .09 | .58 | .56 | .56 | .55 | .56 | .56 | .56 | .56 | .53 |
| 1. Resource Policy_T2_ |  | 5.67 | 1.20 | .76 | .12 | .12 | .55 | .58 | .57 | .56 | .56 | .56 | .56 | .56 | .55 |
| 1. Resource Policy_T3_ |  | 5.46 | 1.27 | .78 | .14 | .16 | .55 | .56 | .61 | .61 | .61 | .62 | .61 | .61 | .60 |
| 1. Resource Policy_T4_ |  | 5.43 | 1.30 | .77 | .15 | .14 | .54 | .54 | .60 | .61 | .62 | .62 | .61 | .62 | .61 |
| 1. Resource Policy_T5_ |  | 5.33 | 1.36 | .79 | .13 | .11 | .54 | .57 | .61 | .62 | .61 | .62 | .61 | .63 | .62 |
| 1. Resource Policy_T6_ |  | 5.18 | 1.34 | .79 | .15 | .14 | .52 | .55 | .60 | .61 | .60 | .62 | .62 | .63 | .62 |
| 1. Resource Policy_T7_ |  | 5.16 | 1.39 | .80 | .16 | .15 | .52 | .55 | .60 | .62 | .61 | .63 | .65 | .65 | .64 |
| 1. Resource Policy_T8_ |  | 4.95 | 1.43 | .81 | .16 | .17 | .53 | .57 | .60 | .62 | .61 | .62 | .64 | .64 | .64 |
| 1. Resource Policy_T9_ |  | 4.92 | 1.45 | .81 | .15 | .18 | .51 | .54 | .59 | .61 | .60 | .62 | .64 | .64 | .66 |
| 1. Symbolic Policy_T1_ |  | 3.28 | 1.42 | .77 | .20 | .23 | .33 | .34 | .36 | .34 | .33 | .34 | .34 | .36 | .36 |
| 1. Symbolic Policy_T2_ |  | 3.42 | 1.45 | .78 | .21 | .25 | .37 | .35 | .38 | .37 | .36 | .36 | .36 | .37 | .39 |
| 1. Symbolic Policy_T3_ |  | 3.04 | 1.40 | .79 | .26 | .29 | .35 | .35 | .40 | .42 | .42 | .42 | .43 | .45 | .44 |
| 1. Symbolic Policy_T4_ |  | 3.17 | 1.43 | .79 | .26 | .27 | .35 | .35 | .43 | .42 | .42 | .43 | .44 | .45 | .45 |
| 1. Symbolic Policy_T5_ |  | 3.02 | 1.40 | .78 | .23 | .21 | .37 | .36 | .42 | .43 | .39 | .41 | .42 | .43 | .44 |
| 1. Symbolic Policy_T6_ |  | 2.95 | 1.31 | .78 | .25 | .22 | .35 | .35 | .43 | .42 | .39 | .39 | .42 | .43 | .44 |
| 1. Symbolic Policy_T7_ |  | 2.93 | 1.34 | .78 | .27 | .22 | .35 | .36 | .41 | .42 | .40 | .40 | .40 | .43 | .43 |
| 1. Symbolic Policy_T8_ |  | 2.83 | 1.32 | .78 | .25 | .20 | .34 | .35 | .40 | .40 | .40 | .40 | .42 | .41 | .42 |
| 1. Symbolic Policy_T9_ |  | 2.74 | 1.34 | .80 | .24 | .21 | .36 | .36 | .42 | .43 | .40 | .40 | .43 | .43 | .43 |

^1^Gender was dummy-coded (0 = Female, 1 = Male); ^2^Age was calculated based on participant’s age at the start of the study (i.e., 2009). Unless noted, *p* < .001. ^ns^*p >* .10; ^+^*p* < .10; ^*^*p* < .05; ^**^*p* < .01

Table S1 (continued). *Descriptive statistics and bivariate correlations for the variables included in this study (ethnic majority group).*

|  |  | 12 | 13 | 14 | 15 | 16 | 17 | 18 | 19 | 20 | 21 | 22 | 23 | 24 | 25 |
| --- | --- | --- | --- | --- | --- | --- | --- | --- | --- | --- | --- | --- | --- | --- | --- |
| 1. Symbolic Exclusion_T1_ |  | -- |  |  |  |  |  |  |  |  |  |  |  |  |  |
| 1. Symbolic Exclusion_T2_ |  | .78 | -- |  |  |  |  |  |  |  |  |  |  |  |  |
| 1. Symbolic Exclusion_T3_ |  | .77 | .82 | -- |  |  |  |  |  |  |  |  |  |  |  |
| 1. Symbolic Exclusion_T4_ |  | .76 | .81 | .82 | -- |  |  |  |  |  |  |  |  |  |  |
| 1. Symbolic Exclusion_T5_ |  | .74 | .79 | .81 | .83 | -- |  |  |  |  |  |  |  |  |  |
| 1. Symbolic Exclusion_T6_ |  | .73 | .78 | .79 | .81 | .82 | -- |  |  |  |  |  |  |  |  |
| 1. Symbolic Exclusion_T7_ |  | .72 | .77 | .78 | .80 | .81 | .83 | -- |  |  |  |  |  |  |  |
| 1. Symbolic Exclusion_T8_ |  | .72 | .75 | .77 | .78 | .79 | .80 | .82 | -- |  |  |  |  |  |  |
| 1. Symbolic Exclusion_T9_ |  | .69 | .73 | .75 | .78 | .78 | .80 | .82 | .83 | -- |  |  |  |  |  |
| 1. Resource Policy_T1_ |  | .52 | .53 | .50 | .53 | .50 | .49 | .48 | .47 | .47 | -- |  |  |  |  |
| 1. Resource Policy_T2_ |  | .50 | .55 | .51 | .54 | .49 | .49 | .49 | .47 | .49 | .78 | -- |  |  |  |
| 1. Resource Policy_T3_ |  | .51 | .56 | .56 | .58 | .54 | .54 | .51 | .51 | .51 | .78 | .81 | -- |  |  |
| 1. Resource Policy_T4_ |  | .49 | .54 | .55 | .57 | .55 | .53 | .51 | .50 | .51 | .76 | .78 | .82 | -- |  |
| 1. Resource Policy_T5_ |  | .50 | .55 | .57 | .59 | .56 | .55 | .53 | .52 | .52 | .76 | .78 | .82 | .84 | -- |
| 1. Resource Policy_T6_ |  | .52 | .56 | .58 | .60 | .56 | .57 | .55 | .53 | .54 | .75 | .77 | .82 | .82 | .84 |
| 1. Resource Policy_T7_ |  | .51 | .56 | .57 | .60 | .57 | .57 | .56 | .55 | .55 | .75 | .77 | .80 | .81 | .83 |
| 1. Resource Policy_T8_ |  | .54 | .58 | .59 | .61 | .59 | .58 | .58 | .58 | .58 | .74 | .76 | .78 | .80 | .82 |
| 1. Resource Policy_T9_ |  | .52 | .56 | .58 | .61 | .58 | .59 | .58 | .58 | .59 | .72 | .75 | .78 | .80 | .81 |
| 1. Symbolic Policy_T1_ |  | .70 | .68 | .67 | .67 | .65 | .65 | .64 | .63 | .62 | .46 | .46 | .45 | .43 | .44 |
| 1. Symbolic Policy_T2_ |  | .67 | .74 | .69 | .70 | .68 | .67 | .67 | .65 | .65 | .46 | .48 | .48 | .46 | .47 |
| 1. Symbolic Policy_T3_ |  | .65 | .69 | .74 | .72 | .71 | .70 | .69 | .68 | .68 | .45 | .45 | .49 | .48 | .50 |
| 1. Symbolic Policy_T4_ |  | .63 | .69 | .71 | .75 | .72 | .70 | .69 | .69 | .69 | .45 | .45 | .49 | .48 | .50 |
| 1. Symbolic Policy_T5_ |  | .63 | .69 | .70 | .72 | .74 | .71 | .70 | .69 | .68 | .44 | .45 | .49 | .47 | .49 |
| 1. Symbolic Policy_T6_ |  | .62 | .68 | .68 | .71 | .70 | .74 | .71 | .69 | .70 | .44 | .45 | .48 | .47 | .47 |
| 1. Symbolic Policy_T7_ |  | .63 | .68 | .68 | .71 | .70 | .72 | .74 | .71 | .72 | .43 | .44 | .46 | .45 | .46 |
| 1. Symbolic Policy_T8_ |  | .61 | .65 | .66 | .68 | .69 | .69 | .71 | .73 | .71 | .42 | .43 | .46 | .44 | .46 |
| 1. Symbolic Policy_T9_ |  | .60 | .65 | .67 | .69 | .69 | .71 | .72 | .73 | .77 | .42 | .43 | .45 | .45 | .47 |

Unless noted, *p* < .001.

Table S1 (continued). *Descriptive statistics and bivariate correlations for the variables included in this study (ethnic majority group).*

|  |  | 26 | 27 | 28 | 29 | 30 | 31 | 32 | 33 | 34 | 35 | 36 | 37 | 38 |
| --- | --- | --- | --- | --- | --- | --- | --- | --- | --- | --- | --- | --- | --- | --- |
| 1. Resource Policy_T6_ |  | -- |  |  |  |  |  |  |  |  |  |  |  |  |
| 1. Resource Policy_T7_ |  | .86 | -- |  |  |  |  |  |  |  |  |  |  |  |
| 1. Resource Policy_T8_ |  | .84 | .87 | -- |  |  |  |  |  |  |  |  |  |  |
| 1. Resource Policy_T9_ |  | .83 | .85 | .87 | -- |  |  |  |  |  |  |  |  |  |
| 1. Symbolic Policy_T1_ |  | .46 | .46 | .48 | .46 | -- |  |  |  |  |  |  |  |  |
| 1. Symbolic Policy_T2_ |  | .48 | .49 | .51 | .49 | .81 | -- |  |  |  |  |  |  |  |
| 1. Symbolic Policy_T3_ |  | .51 | .51 | .53 | .51 | .79 | .83 | -- |  |  |  |  |  |  |
| 1. Symbolic Policy_T4_ |  | .52 | .53 | .54 | .53 | .78 | .82 | .83 | -- |  |  |  |  |  |
| 1. Symbolic Policy_T5_ |  | .49 | .50 | .51 | .51 | .77 | .81 | .82 | .85 | -- |  |  |  |  |
| 1. Symbolic Policy_T6_ |  | .50 | .51 | .52 | .52 | .77 | .81 | .81 | .83 | .84 | -- |  |  |  |
| 1. Symbolic Policy_T7_ |  | .49 | .51 | .52 | .51 | .77 | .80 | .81 | .82 | .82 | .85 | -- |  |  |
| 1. Symbolic Policy_T8_ |  | .48 | .50 | .53 | .52 | .74 | .77 | .79 | .80 | .81 | .83 | .85 | -- |  |
| 1. Symbolic Policy_T9_ |  | .49 | .51 | .52 | .54 | .73 | .76 | .78 | .80 | .80 | .82 | .84 | .85 | -- |

Unless noted, *p* < .001.

Table S2. *Descriptive statistics and bivariate correlations for the variables included in this study (ethnic minority group).*

|  |  | *Mean* | *SD* | α | 1 | 2 | 3 | 4 | 5 | 6 | 7 | 8 | 9 | 10 | 11 |
| --- | --- | --- | --- | --- | --- | --- | --- | --- | --- | --- | --- | --- | --- | --- | --- |
| 1. Gender^1^ |  | 0.36 | 0.48 | --- | -- |  |  |  |  |  |  |  |  |  |  |
| 1. Age^2^ |  | 39.27 | 13.79 | --- | .11 | -- |  |  |  |  |  |  |  |  |  |
| 1. Historical Negation_T1_ |  | 4.93 | 1.54 | .76 | -.02^ns^ | .05^*^ | -- |  |  |  |  |  |  |  |  |
| 1. Historical Negation_T2_ |  | 4.86 | 1.55 | .78 | -.00^ns^ | .08^**^ | .69 | -- |  |  |  |  |  |  |  |
| 1. Historical Negation_T3_ |  | 4.73 | 1.58 | .81 | .03^ns^ | .07^**^ | .72 | .72 | -- |  |  |  |  |  |  |
| 1. Historical Negation_T4_ |  | 4.77 | 1.55 | .79 | .00^ns^ | .03^ns^ | .68 | .71 | .75 | -- |  |  |  |  |  |
| 1. Historical Negation_T5_ |  | 4.68 | 1.58 | .78 | .04^*^ | .04^*^ | .68 | .73 | .75 | .73 | -- |  |  |  |  |
| 1. Historical Negation_T6_ |  | 4.62 | 1.55 | .80 | .03^ns^ | .08 | .66 | .70 | .72 | .71 | .75 | -- |  |  |  |
| 1. Historical Negation_T7_ |  | 4.53 | 1.60 | .80 | .08 | .06^**^ | .69 | .68 | .71 | .71 | .72 | .77 | -- |  |  |
| 1. Historical Negation_T8_ |  | 4.55 | 1.65 | .80 | .08 | .07 | .66 | .68 | .70 | .68 | .72 | .76 | .78 | -- |  |
| 1. Historical Negation_T9_ |  | 4.45 | 1.68 | .81 | .11 | .10 | .64 | .67 | .70 | .69 | .70 | .73 | .75 | .77 | -- |
| 1. Symbolic Exclusion_T1_ |  | 2.84 | 1.45 | .72 | .12 | .09 | .38 | .40 | .42 | .41 | .41 | .42 | .47 | .49 | .48 |
| 1. Symbolic Exclusion_T2_ |  | 2.75 | 1.41 | .75 | .16 | .10 | .41 | .42 | .45 | .45 | .40 | .44 | .45 | .48 | .49 |
| 1. Symbolic Exclusion_T3_ |  | 2.58 | 1.37 | .78 | .16 | .11 | .39 | .43 | .44 | .44 | .43 | .48 | .48 | .51 | .51 |
| 1. Symbolic Exclusion_T4_ |  | 2.66 | 1.42 | .79 | .13 | .11 | .40 | .40 | .44 | .41 | .42 | .46 | .47 | .49 | .48 |
| 1. Symbolic Exclusion_T5_ |  | 2.55 | 1.38 | .78 | .14 | .06 | .41 | .43 | .47 | .42 | .42 | .44 | .49 | .50 | .48 |
| 1. Symbolic Exclusion_T6_ |  | 2.51 | 1.35 | .77 | .14 | .07 | .40 | .37 | .42 | .41 | .40 | .42 | .47 | .48 | .45 |
| 1. Symbolic Exclusion_T7_ |  | 2.43 | 1.34 | .78 | .14 | .10 | .40 | .40 | .44 | .42 | .43 | .42 | .47 | .50 | .47 |
| 1. Symbolic Exclusion_T8_ |  | 2.37 | 1.33 | .78 | .12 | .10 | .35 | .36 | .43 | .41 | .41 | .41 | .45 | .45 | .45 |
| 1. Symbolic Exclusion_T9_ |  | 2.31 | 1.32 | .81 | .17 | .13 | .38 | .40 | .46 | .40 | .42 | .42 | .45 | .47 | .46 |
| 1. Resource Policy_T1_ |  | 4.26 | 1.71 | .82 | .03^ns^ | .03^ns^ | .42 | .44 | .44 | .46 | .45 | .44 | .47 | .45 | .46 |
| 1. Resource Policy_T2_ |  | 4.31 | 1.72 | .84 | .05^ns^ | .00^ns^ | .44 | .46 | .47 | .49 | .46 | .43 | .48 | .47 | .46 |
| 1. Resource Policy_T3_ |  | 4.35 | 1.69 | .84 | .10 | .05^†^ | .45 | .48 | .49 | .53 | .51 | .50 | .53 | .54 | .54 |
| 1. Resource Policy_T4_ |  | 4.00 | 1.72 | .83 | .07 | .03^†^ | .49 | .47 | .52 | .45 | .44 | .44 | .50 | .51 | .49 |
| 1. Resource Policy_T5_ |  | 4.07 | 1.75 | .84 | .10 | -.00^ns^ | .46 | .47 | .49 | .48 | .46 | .48 | .54 | .52 | .53 |
| 1. Resource Policy_T6_ |  | 4.01 | 1.69 | .84 | .12 | .00^ns^ | .47 | .47 | .52 | .49 | .49 | .48 | .54 | .54 | .52 |
| 1. Resource Policy_T7_ |  | 3.96 | 1.71 | .84 | .12 | .02^ns^ | .46 | .51 | .51 | .51 | .53 | .53 | .54 | .55 | .53 |
| 1. Resource Policy_T8_ |  | 3.84 | 1.69 | .84 | .13 | .06 | .47 | .51 | .51 | .51 | .51 | .52 | .55 | .53 | .55 |
| 1. Resource Policy_T9_ |  | 3.93 | 1.68 | .83 | .15 | .09 | .48 | .48 | .52 | .49 | .50 | .51 | .57 | .54 | .54 |
| 1. Symbolic Policy_T1_ |  | 2.58 | 1.28 | .74 | .10 | .09 | .23 | .26 | .26 | .31 | .26 | .26 | .31 | .30 | .31 |
| 1. Symbolic Policy_T2_ |  | 2.66 | 1.31 | .76 | .12 | .06^*^ | .28 | .28 | .32 | .30 | .30 | .31 | .34 | .35 | .38 |
| 1. Symbolic Policy_T3_ |  | 2.49 | 1.24 | .75 | .17 | .13 | .26 | .26 | .30 | .33 | .32 | .33 | .38 | .40 | .38 |
| 1. Symbolic Policy_T4_ |  | 2.49 | 1.25 | .74 | .13 | .11 | .32 | .30 | .34 | .26 | .29 | .30 | .34 | .38 | .33 |
| 1. Symbolic Policy_T5_ |  | 2.47 | 1.26 | .75 | .14 | .07 | .29 | .27 | .33 | .32 | .28 | .31 | .37 | .37 | .36 |
| 1. Symbolic Policy_T6_ |  | 2.44 | 1.19 | .74 | .15 | .07 | .25 | .23 | .32 | .31 | .28 | .27 | .33 | .36 | .35 |
| 1. Symbolic Policy_T7_ |  | 2.40 | 1.20 | .75 | .16 | .08 | .26 | .26 | .34 | .28 | .31 | .31 | .32 | .36 | .36 |
| 1. Symbolic Policy_T8_ |  | 2.36 | 1.16 | .73 | .16 | .10 | .24 | .23 | .31 | .32 | .32 | .32 | .33 | .32 | .34 |
| 1. Symbolic Policy_T9_ |  | 2.35 | 1.17 | .74 | .18 | .12 | .23 | .24 | .33 | .27 | .30 | .30 | .33 | .35 | .33 |

^1^Gender was dummy-coded (0 = Female, 1 = Male); ^2^Age was calculated based on participant’s age at the start of the study (i.e., 2009). Unless noted, *p* < .001. ^ns^*p >* .10; ^†^*p* < .10; ^*^*p* < .05; ^**^*p* < .01

Table S2 (continued). *Descriptive statistics and bivariate correlations for the variables included in this study (ethnic minority group).*

|  |  | 12 | 13 | 14 | 15 | 16 | 17 | 18 | 19 | 20 | 21 | 22 | 23 | 24 | 25 |
| --- | --- | --- | --- | --- | --- | --- | --- | --- | --- | --- | --- | --- | --- | --- | --- |
| 1. Symbolic Exclusion_T1_ |  | -- |  |  |  |  |  |  |  |  |  |  |  |  |  |
| 1. Symbolic Exclusion_T2_ |  | .71 | -- |  |  |  |  |  |  |  |  |  |  |  |  |
| 1. Symbolic Exclusion_T3_ |  | .71 | .74 | -- |  |  |  |  |  |  |  |  |  |  |  |
| 1. Symbolic Exclusion_T4_ |  | .70 | .74 | .78 | -- |  |  |  |  |  |  |  |  |  |  |
| 1. Symbolic Exclusion_T5_ |  | .69 | .73 | .76 | .78 | -- |  |  |  |  |  |  |  |  |  |
| 1. Symbolic Exclusion_T6_ |  | .67 | .69 | .73 | .75 | .75 | -- |  |  |  |  |  |  |  |  |
| 1. Symbolic Exclusion_T7_ |  | .63 | .69 | .72 | .73 | .77 | .77 | -- |  |  |  |  |  |  |  |
| 1. Symbolic Exclusion_T8_ |  | .64 | .68 | .72 | .72 | .74 | .75 | .76 | -- |  |  |  |  |  |  |
| 1. Symbolic Exclusion_T9_ |  | .60 | .67 | .69 | .70 | .74 | .74 | .76 | .78 | -- |  |  |  |  |  |
| 1. Resource Policy_T1_ |  | .49 | .45 | .45 | .48 | .45 | .45 | .43 | .42 | .38 | -- |  |  |  |  |
| 1. Resource Policy_T2_ |  | .45 | .46 | .44 | .48 | .45 | .43 | .42 | .42 | .38 | .84 | -- |  |  |  |
| 1. Resource Policy_T3_ |  | .45 | .48 | .48 | .50 | .47 | .45 | .45 | .43 | .40 | .84 | .87 | -- |  |  |
| 1. Resource Policy_T4_ |  | .50 | .48 | .50 | .52 | .48 | .46 | .45 | .44 | .43 | .83 | .86 | .86 | -- |  |
| 1. Resource Policy_T5_ |  | .44 | .48 | .49 | .54 | .54 | .49 | .51 | .48 | .47 | .81 | .85 | .84 | .85 | -- |
| 1. Resource Policy_T6_ |  | .47 | .50 | .51 | .54 | .55 | .52 | .51 | .50 | .48 | .82 | .84 | .84 | .85 | .87 |
| 1. Resource Policy_T7_ |  | .48 | .50 | .53 | .55 | .56 | .52 | .54 | .50 | .49 | .81 | .83 | .84 | .83 | .87 |
| 1. Resource Policy_T8_ |  | .49 | .50 | .53 | .56 | .58 | .55 | .56 | .54 | .55 | .78 | .81 | .81 | .83 | .84 |
| 1. Resource Policy_T9_ |  | .45 | .48 | .54 | .54 | .58 | .55 | .56 | .55 | .56 | .76 | .80 | .80 | .80 | .83 |
| 1. Symbolic Policy_T1_ |  | .60 | .56 | .54 | .58 | .56 | .57 | .50 | .54 | .53 | .50 | .47 | .48 | .47 | .47 |
| 1. Symbolic Policy_T2_ |  | .55 | .61 | .56 | .58 | .60 | .58 | .55 | .54 | .53 | .47 | .49 | .50 | .46 | .50 |
| 1. Symbolic Policy_T3_ |  | .54 | .58 | .65 | .63 | .63 | .63 | .61 | .63 | .58 | .47 | .47 | .50 | .47 | .50 |
| 1. Symbolic Policy_T4_ |  | .53 | .57 | .62 | .67 | .61 | .58 | .58 | .57 | .57 | .45 | .46 | .45 | .52 | .49 |
| 1. Symbolic Policy_T5_ |  | .53 | .57 | .60 | .65 | .66 | .60 | .62 | .60 | .61 | .44 | .45 | .46 | .47 | .53 |
| 1. Symbolic Policy_T6_ |  | .52 | .55 | .59 | .62 | .61 | .64 | .63 | .63 | .61 | .42 | .44 | .44 | .44 | .47 |
| 1. Symbolic Policy_T7_ |  | .46 | .53 | .60 | .62 | .62 | .63 | .66 | .63 | .64 | .41 | .41 | .45 | .46 | .49 |
| 1. Symbolic Policy_T8_ |  | .51 | .53 | .60 | .60 | .61 | .60 | .62 | .64 | .62 | .37 | .39 | .42 | .43 | .46 |
| 1. Symbolic Policy_T9_ |  | .47 | .52 | .58 | .59 | .62 | .62 | .64 | .65 | .69 | .36 | .36 | .39 | .41 | .44 |

Unless noted, *p* < .001.

Table S2 (continued). *Descriptive statistics and bivariate correlations for the variables included in this study (ethnic minority group).*

|  |  | 26 | 27 | 28 | 29 | 30 | 31 | 32 | 33 | 34 | 35 | 36 | 37 | 38 |
| --- | --- | --- | --- | --- | --- | --- | --- | --- | --- | --- | --- | --- | --- | --- |
| 1. Resource Policy_T6_ |  | -- |  |  |  |  |  |  |  |  |  |  |  |  |
| 1. Resource Policy_T7_ |  | .88 | -- |  |  |  |  |  |  |  |  |  |  |  |
| 1. Resource Policy_T8_ |  | .86 | .88 | -- |  |  |  |  |  |  |  |  |  |  |
| 1. Resource Policy_T9_ |  | .84 | .87 | .87 | -- |  |  |  |  |  |  |  |  |  |
| 1. Symbolic Policy_T1_ |  | .48 | .46 | .48 | .46 | -- |  |  |  |  |  |  |  |  |
| 1. Symbolic Policy_T2_ |  | .51 | .49 | .51 | .49 | .75 | -- |  |  |  |  |  |  |  |
| 1. Symbolic Policy_T3_ |  | .50 | .52 | .51 | .49 | .76 | .78 | -- |  |  |  |  |  |  |
| 1. Symbolic Policy_T4_ |  | .50 | .51 | .50 | .50 | .71 | .76 | .75 | -- |  |  |  |  |  |
| 1. Symbolic Policy_T5_ |  | .51 | .51 | .52 | .51 | .72 | .76 | .79 | .77 | -- |  |  |  |  |
| 1. Symbolic Policy_T6_ |  | .52 | .49 | .51 | .52 | .71 | .73 | .77 | .74 | .79 | -- |  |  |  |
| 1. Symbolic Policy_T7_ |  | .50 | .54 | .53 | .54 | .67 | .71 | .76 | .76 | .78 | .81 | -- |  |  |
| 1. Symbolic Policy_T8_ |  | .48 | .49 | .53 | .50 | .67 | .69 | .76 | .72 | .75 | .78 | .81 | -- |  |
| 1. Symbolic Policy_T9_ |  | .46 | .47 | .50 | .53 | .65 | .69 | .74 | .71 | .76 | .77 | .80 | .79 | -- |

Unless noted, *p* < .001.

1. Because both New Zealand citizens and permanent residents who are 18 years of age or older are eligible to vote in New Zealand, randomly sampling from the electoral roll is as close to a random sample of the adult population as one can get. [↑](#footnote-ref-1)
